# Catalytic promiscuity of *O*-methyltransferases from *Corydalis yanhusuo* leading to the structural diversity of benzylisoquinoline alkaloids

**DOI:** 10.1093/hr/uhac152

**Published:** 2022-07-06

**Authors:** Junling Bu, Xiuhua Zhang, Qishuang Li, Ying Ma, Zhimin Hu, Jian Yang, Xiuyu Liu, Ruishan Wang, Xiang Jiao, Tong Chen, Changjiangsheng Lai, Guanghong Cui, Jinfu Tang, Yu Kong, Lei Yang, Sheng Lin, Yun Chen, Juan Guo, Luqi Huang

**Affiliations:** State Key Laboratory of Dao-di Herbs, National Resource Center for Chinese Materia Medica, China Academy of Chinese Medical Sciences, No. 16 South Side Street, Dongzhimen, Beijing 100700, China; State Key Laboratory of Dao-di Herbs, National Resource Center for Chinese Materia Medica, China Academy of Chinese Medical Sciences, No. 16 South Side Street, Dongzhimen, Beijing 100700, China; State Key Laboratory of Dao-di Herbs, National Resource Center for Chinese Materia Medica, China Academy of Chinese Medical Sciences, No. 16 South Side Street, Dongzhimen, Beijing 100700, China; State Key Laboratory of Dao-di Herbs, National Resource Center for Chinese Materia Medica, China Academy of Chinese Medical Sciences, No. 16 South Side Street, Dongzhimen, Beijing 100700, China; State Key Laboratory of Dao-di Herbs, National Resource Center for Chinese Materia Medica, China Academy of Chinese Medical Sciences, No. 16 South Side Street, Dongzhimen, Beijing 100700, China; State Key Laboratory of Dao-di Herbs, National Resource Center for Chinese Materia Medica, China Academy of Chinese Medical Sciences, No. 16 South Side Street, Dongzhimen, Beijing 100700, China; School of Pharmacy, Henan University of Chinese Medicine, No. 156 Jinshuidong Road, Zhengzhou 450008, China; State Key Laboratory of Dao-di Herbs, National Resource Center for Chinese Materia Medica, China Academy of Chinese Medical Sciences, No. 16 South Side Street, Dongzhimen, Beijing 100700, China; Department of Biology and Biological Engineering, Chalmers University of Technology, Kemivägen 10, SE41296, Gothenburg, Sweden; State Key Laboratory of Dao-di Herbs, National Resource Center for Chinese Materia Medica, China Academy of Chinese Medical Sciences, No. 16 South Side Street, Dongzhimen, Beijing 100700, China; State Key Laboratory of Dao-di Herbs, National Resource Center for Chinese Materia Medica, China Academy of Chinese Medical Sciences, No. 16 South Side Street, Dongzhimen, Beijing 100700, China; State Key Laboratory of Dao-di Herbs, National Resource Center for Chinese Materia Medica, China Academy of Chinese Medical Sciences, No. 16 South Side Street, Dongzhimen, Beijing 100700, China; State Key Laboratory of Dao-di Herbs, National Resource Center for Chinese Materia Medica, China Academy of Chinese Medical Sciences, No. 16 South Side Street, Dongzhimen, Beijing 100700, China; Shanghai Key Laboratory of Plant Functional Genomics and Resources, Shanghai Chenshan Botanical Garden, Shanghai, 201602, China; Shanghai Key Laboratory of Plant Functional Genomics and Resources, Shanghai Chenshan Botanical Garden, Shanghai, 201602, China; Key Laboratory of Chinese Internal Medicine of Ministry of Education and Beijing, Dongzhimen Hospital, Beijing University of Chinese Medicine, Beijing 100700, China; Department of Biology and Biological Engineering, Chalmers University of Technology, Kemivägen 10, SE41296, Gothenburg, Sweden; State Key Laboratory of Dao-di Herbs, National Resource Center for Chinese Materia Medica, China Academy of Chinese Medical Sciences, No. 16 South Side Street, Dongzhimen, Beijing 100700, China; State Key Laboratory of Dao-di Herbs, National Resource Center for Chinese Materia Medica, China Academy of Chinese Medical Sciences, No. 16 South Side Street, Dongzhimen, Beijing 100700, China

## Abstract

*O*-methyltransferases play essential roles in producing structural diversity and improving the biological properties of benzylisoquinoline alkaloids (BIAs) in plants. In this study, *Corydalis yanhusuo*, a plant used in traditional Chinese medicine due to the analgesic effects of its BIA-active compounds, was employed to analyze the catalytic characteristics of *O*-methyltransferases in the formation of BIA diversity. Seven genes encoding *O*-methyltransferases were cloned, and functionally characterized using seven potential BIA substrates. Specifically, an *O*-methyltransferase (CyOMT2) with highly efficient catalytic activity of both 4′- and 6-*O*-methylations of 1-BIAs was found. CyOMT6 was found to perform two sequential methylations at both 9- and 2-positions of the essential intermediate of tetrahydroprotoberberines, (*S*)-scoulerine. Two *O*-methyltransferases (CyOMT5 and CyOMT7) with wide substrate promiscuity were found, with the 2-position of tetrahydroprotoberberines as the preferential catalytic site for CyOMT5 (named scoulerine 2-*O*-methyltransferase) and the 6-position of 1-BIAs as the preferential site for CyOMT7. In addition, results of integrated phylogenetic molecular docking analysis and site-directed mutation suggested that residues at sites 172, 306, 313, and 314 in CyOMT5 are important for enzyme promiscuity related to *O*-methylations at the 6- and 7-positions of isoquinoline. Cys at site 253 in CyOMT2 was proved to promote the methylation activity of the 6-position and to expand substrate scopes. This work provides insight into *O*-methyltransferases in producing BIA diversity in *C. yanhusuo* and genetic elements for producing BIAs by metabolic engineering and synthetic biology.

## Introduction

**Figure 1:**
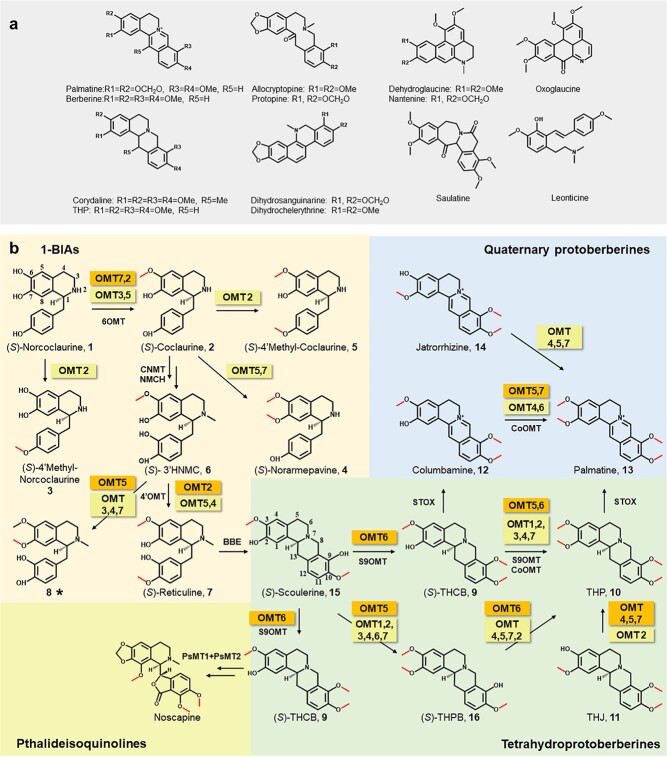
Chemical structures of alkaloids in *C. yanhusuo* and contributions of *OMT*s to BIA biosynthesis. **a** Structural types and representative compounds of *C. yanhusuo*. **b** Proposed *O*-methylation routes involved in BIA biosynthesis in *C. yanhusuo* based on the detected functions of CyOMT1–7 (abbreviated as OMT1–7). These enzymes are highlighted in orange or yellow, representing reactions they catalyze with relatively high or low efficiency, respectively. Enzymes that were not characterized in this study but that catalyze these reactions are shown without highlighting. A new product induced by CyOMT5 is marked with an asterisk. Double arrows indicate multistep reactions and single arrows indicate one-step reactions. Abbreviations: 6OMT, norcoclaurine 6-*O*-methyltransferase; CNMT, (*S*)-coclaurine-*N*-methyltransferase; NMCH, (*S*)-*N*-methylcoclaurine 3′-hydroxylase; 4′OMT, 3′-hydroxy-*N*-methylcoclaurine 4′-*O*-methyltransferase; BBE, berberine bridge enzyme; S9OMT, (*S*)-scoulerine 9-*O*-methyltransferase; CoOMT, columbamine *O*-methyltransferase; STOX, (*S*)-tetrahydroprotoberberine oxidase; THCB, tetrahydrocolumbamine (9); THPB, tetrahydropalmatrubine (16); THP, tetrahydropalmatine (10); THJ, tetrahydrojatrorrhizine (11); (*S*)-3′HNMC, (*S*)-3′-hydroxy-*N*-methylcoclaurine (6).

Benzylisoquinoline alkaloids (BIAs) are a diverse group of specialized plant metabolites comprising ~2500 known structures primarily found in four plant families: Papaveraceae, Ranunculaceae, Berberidaceae, and Menispermaceae [1]. Many medically relevant compounds are BIAs, including codeine and morphine from opium poppy (*Papaver somniferum*), berberine from plants in the *Coptis* genus, and tetrahydropalmatine [10] from *Corydalis yanhusuo*. BIAs accumulate in plants as secondary metabolites, sometimes with low concentration or with abundant derivatives; for example, ~100 000 hectares of opium poppy are cultivated annually to meet legal medical and scientific demand [2]. Production of such medically important compounds by synthetic biology is therefore an attractive alternative. Some compounds, such as morphine, thebaine, noscapine, tetrahydropalmatine (compound **10** in Fig. 1), dihydrosanguinarine, and canadine can already be generated biosynthetically [3–7]. Furthermore, researchers have leveraged the known catalytic promiscuity of some post-modification enzymes in BIA biosynthetic pathways to produce new compounds [8]. A thorough understanding of the genetics underlying BIA biosynthesis and characterization of the enzymes in these pathways thus remains an important area of research.

*C. yanhusuo*, which is rich in BIA alkaloids, has been in widespread use over thousands of years in traditional Chinese medicine to promote blood circulation and reinforce vital energy as well as to alleviate pains in many conditions, such as stomach ache, dysmenorrhea, and traumatic injury [9]. It has been reported that tetrahydropalmatine, corydalis, and protopine, which are abundant in *C. yanhusuo*, are mainly responsible for its analgesic effects [10]. Studies with animal models of substance use disorder (SUD) and target research indicated that tetrahydroprotoberberines are attractive candidates for treating SUD, including the treatment of misuse of opiates and other psychotropic drugs [11–14]. *C. yanhusuo* is also one of the components of the JiTai tablet, a Chinese patent drug for detoxification and anti-addiction treatment [15, 16]. In addition to the medicinal importance of the compounds mentioned above, *C. yanhusuo* contains more than 60 alkaloids. The majority are tetrahydroprotoberberines and quaternary protoberberines, but most of the others have been classified as aporphines, protopines, or benzophenanthridines based on their chemical structures (Fig. 1a) [17]. The sheer abundance of BIAs in *C. yanhusuo* makes it an ideal candidate for investigation of the genetic elements contributing to the formation of BIA diversity.

Methylation of natural products has been reported to affect the chemical properties or to change biological activity by adding hydrophobic methyl groups [18]. In BIA biosynthetic pathways, *O*-methyltransferases (OMTs) play an important role in the functionalization of specialized metabolites, specifically 1-benzylisoquinolines (1-BIAs), tetrahydroprotoberberines, quaternary protoberberines, and phthalideisoquinolines (Fig. 1b) [19]. OMTs known to be involved in BIA biosynthesis have been isolated mainly from the genera *Papaver* and *Coptis*, and include those involved in the central biosynthetic pathway and post-modification process [19–27]. The catalytic promiscuity of OMTs contributes to a complex metabolic network and provides a basis for the production of new compounds [20, 21]. This feature of OMTs provides a foundation for producing diversified BIAs. Thus, a thorough characterization of OMTs and the structure–function correlation could provide a reference for future protein engineering and synthetic biology utilization.

To gain further insight into OMTs in the formation of BIA diversity, a transcriptome was generated for *C. yanhusuo* and seven OMTs were selected for functional characterization. Seven potential BIAs representing the three main BIA types were selected as substrates to analyze the catalytic promiscuity of OMTs. Notably, we obtained new functional OMTs that could selectively and efficiently catalyze methylation of position C7 of (*S*)-3′-hydroxy-*N*-methylcoclaurine (**6**) and specifically catalyze position C2 of (*S*)-scoulerine (**15**). The catalytic promiscuity of CyOMTs leads to structural diversity of BIAs and new product structures. Furthermore, 13 functionally known OMTs were employed as examples for structure–function analysis based on phylogenetic analysis and molecular docking, leading to identification of critical residues for OMT catalytic preference. Our work contributes to the comprehensive investigation of the BIA biosynthetic network and characterization of the related post-modification enzymes. This is expected to provide valuable insight into the genetic elements required for heterologous production of these compounds and for novel compound production.

## Results

### Transcriptome analysis and identification of *O*-methyltransferases in *C. yanhusuo*

*C. yanhusuo* is rich in BIA alkaloids**,** including mainly tetrahydroprotoberberines and quaternary protoberberines, as well as aporphines, protopines, and benzophenanthridines. Abundance of *O*-methylated derivatives promotes the diversity of BIAs in *C. yanhusuo* and makes it an ideal candidate for investigating the genetic elements involved in BIA biosynthesis. To investigate *OMT*s involved in BIA biosynthesis in *C. yanhusuo*, RNA from the bulb and leaf, which accumulate different kinds of BIAs, were extracted and utilized for Illumina paired-end cDNA library construction and sequencing. Briefly, a non-normalized cDNA library was prepared from different tissues of *C. yanhusuo* and sequenced using on an Illumina NovaSeq 6000 platform. The analysis of sequencing data quality is listed in Supplementary Data Table S1. The Trinity *de novo* assembler found 96 182 transcripts ranging from 201 to 16 082 bp with the mean length of 1265 bp (accession number: RPJCA, CRA005108). Annotation information revealed 253 genes annotated as ‘*O*-Methyltransferase’ in the transcriptome of *C. yanhusuo*. Twenty-eight of these were selected as candidate OMTs based on their FPKM (fragments per kilobase of transcript per million mapped reads) value >50. Of these, seven genes with full open reading frames were cloned successfully (sequences and primers of CyOMT1–CyOMT7 are shown in Supplementary Data Tables S2 and S3, respectively).

### Phylogenetic analysis of *Corydalis yanhusuo O*-methyltransferases

**Figure 2:**
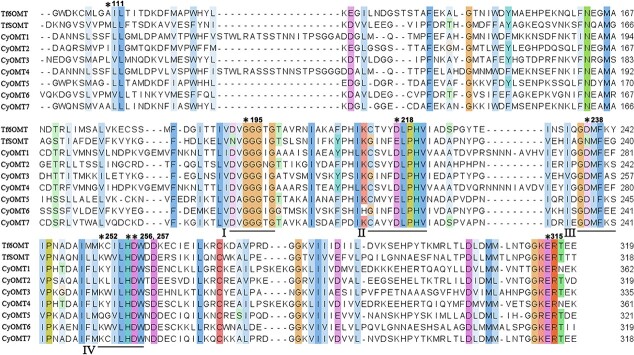
Multiple sequence alignment of CyOMTs, *T. flavum* 6OMT (Tf6OMT) and *T. flavum* SOMT (TfSOMT). Asterisks indicate conserved residues, namely BIA binding sites (H256, D257), SAM binding sites (G195, D218, D238, K252), a critical residue (E315), and residue M111 in TfSOMT. Conserved motifs I–IV are underlined.

Multiple amino acid sequence alignment showed high conservation of key residues in the seven cloned OMTs. These included the *S*-adenosylhomocysteine/S-adenosylmethionine (SAH/SAM) interaction region and four conserved motifs (labeled I–IV) characteristic of the SAM-binding site in plant SAM-dependent methyltransferases [28]. The residues within these motifs that directly interact with SAM, including G195 in motif I, D218 in motif II, D238 in motif III, and K252 in motif IV, were conserved among the seven CyOMTs examined, with the exception of D218E and K252Q substitutions in CyOMT5. The residues involved in BIA substrate binding, H256 and D257, and the key catalytic residue E315 were also conserved among the seven CyOMTs [29]. Furthermore, the highly conserved glycine-rich ‘GxGxG’ sequence in SAM-binding motif I was found in CyOMT1–CyOMT7. It has been shown that M111 of *Thalictrum flavum* S9OMT is a key residue for 9-*O*-methylation of scoulerine (**15**), and that mutation of M111 to alanine results in an expanded substrate scope toward various 1-BIA substrates [5]. Here, CyOMT6 catalyzing both 9- and 2-positions of the essential intermediate of tetrahydroprotoberberines was the only OMT examined to have the M111 residue (see below, Fig. 2).

**Figure 3:**
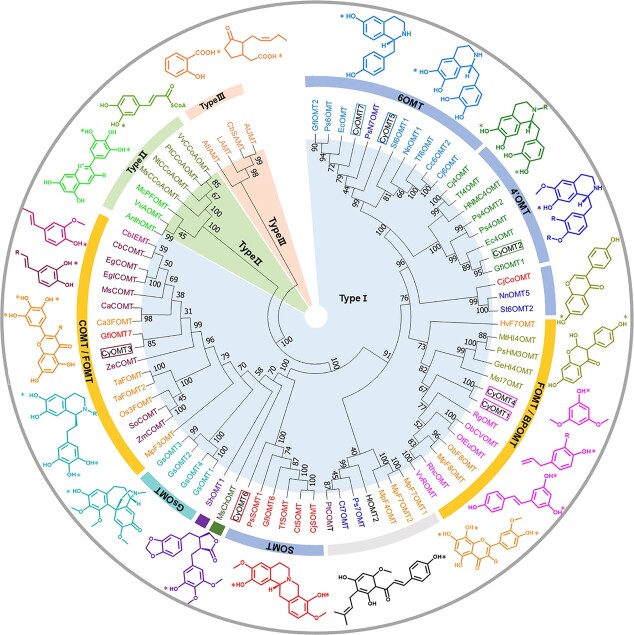
Phylogenetic relationships among OMTs in *C. yanhusuo.* OMTs include CyOMTs characterized in this study (enzyme names outlined) and previously characterized OMTs in other species. Molecular structures of representative substrates are shown at the outer edge, with catalytic sites indicated by asterisks. Enzyme names and corresponding substrates are shown in the same color. Abbreviations: 6OMT, norcoclaurine 6-*O*-methyltransferase; 4′OMT, 3′-hydroxy-*N*-methylcoclaurine 4′-*O*-methyltransferase; COMT, caffeic acid *O*-methyltransferase; FOMT, flavanone *O*-methyltransferase; BPOMT, benzenoid phenolics *O*-methyltransferase; SOMT, scoulerine 9-*O*-methyltransferase; GsOMT, *Gloriosa superba O*-methyltransferase. Abbreviations and accessions numbers for each enzyme are shown in Supplementary Data Table S4.

The relationship between OMT phylogeny and substrate preference was analyzed using seven successfully cloned OMTs and 68 functionally characterized plant OMTs, including those involved in synthesis of BIAs, flavonoids, phenylpropanoids, and benzenoid phenolics. In the nomenclature proposed by Noel *et al*. [30], plant OMTs can be categorized as type I, type II, or type III. Phylogenetic analysis here revealed that type III OMTs participate in converting carboxylic acids to methyl ester derivatives, such as salicylic acid carboxyl methyltransferase (SAMT) and jasmonic acid carboxyl methyltransferase (JMT) involved in phytohormone biosynthesis, and indole-3-acetate *O*-methyltransferase (IAMT) and loganic acid methyltransferase (LAMT) involved in alkaloid biosynthesis. Type II OMTs represent a group of lower molecular mass and cation-dependent OMTs, which are specific for coenzyme A-derived phenylpropanoid compounds, and some are involved in anthocyanin and phenylpropanoid metabolism. Type I OMTs were more diversified and primarily involved in specific metabolic pathways in plants, including those of caffeic acid, benzenoid phenolics, flavonoids, and alkaloids. Phylogenetic analysis clustered most of the alkaloid catalytic OMTs of BIAs (in the blue section of the innermost ring in Fig. 3) and colchicine (in the green section of the innermost ring) are clustered separately. This provides a foundation for substrate selection and functional characterization of OMTs. It shows that CyOMT2, CyOMT5, CyOMT6, and CyOMT7 cluster with OMTs involved in biosynthesis of BIAs, while CyOMT1, CyOMT3, and CyOMT4 cluster with those involved in biosynthesis of flavonoids, phenylpropanoids, or benzenoid phenolics.

### Functional characterization of *Corydalis yanhusuo O*-methyltransferases *in vitro*

Full-length cDNA for each of the seven CyOMT candidate genes was cloned into the pET32a vector and expressed in *Escherichia coli* BL21 (Supplementary Data Fig. S1a). Recombinant expressed proteins were purified for *in vitro* assays (Supplementary Data Fig. S1b). *C. yanhusuo* mainly accumulates tetrahydroprotoberberine and quaternary protoberberine alkaloids, and 1-benzylisoquinoline has been recognized as the common intermediate in biosynthetically characterized BIAs. Thus, the seven BIAs selected as candidate substrates represent the 1-BIA, tetrahydroprotoberberine, and quaternary protoberberine alkaloids with hydroxylation groups for further methylation. The included 1-BIAs contained hydroxyl groups at C6, C7, C3′, and C4′, and tetrahydroprotoberberine/quaternary protoberberine alkaloids had hydroxyl groups at C2, C3, and C9, providing abundant sites for methylation. The catalytic characteristics of these OMTs were analyzed by *in vitro* assay and detected by UPLC–TOF–MS for qualitative analysis and by UPLC for quantitative analysis.

**Figure 4:**
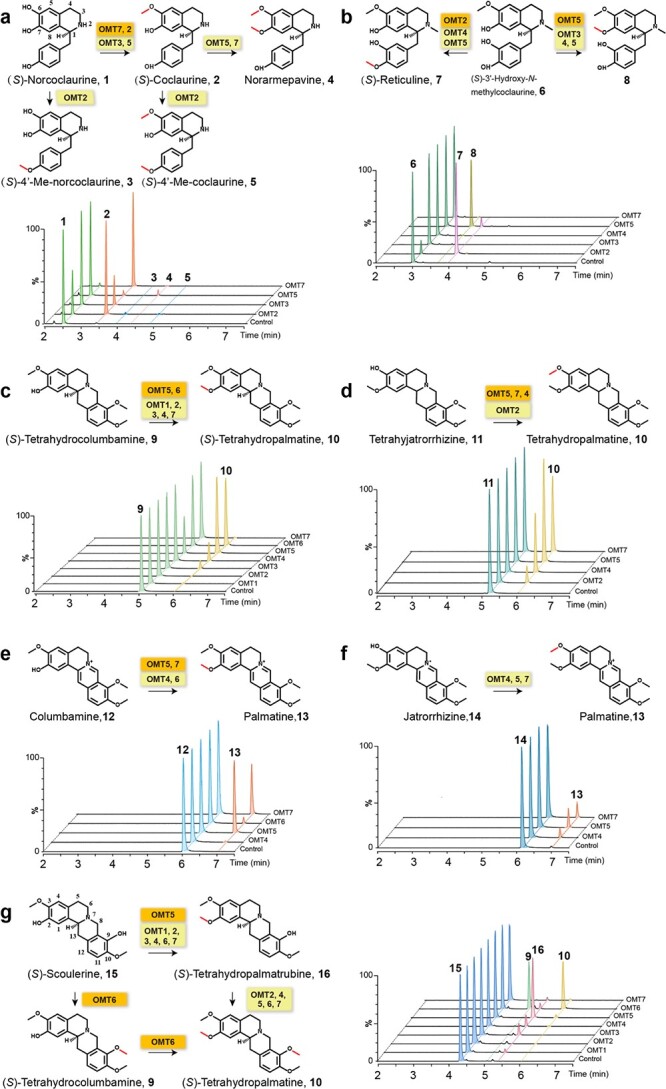
Methylation catalyzed by CyOMTs. Substrates and products of CyOMT reactions are shown. Reaction products were determined as the addition of 14, 28, or 42 Da to the molecular ion of each possible substrate, and chromatographic and mass spectrometric data were compared with the available authentic standards shown in Supplementary Data Table S5 or published CID analysis. Functional genes of CyOMT1–7 (abbreviated OMT1–7) are marked above the arrow in the catalytic diagram. These enzymes are highlighted in orange or yellow for reactions they catalyze with relatively high or low efficiency. Numbers above peaks correspond to the number marked behind the substrate and product in the catalytic diagrams. The peaks of the same compound are filled with the same color. **a** [M + H]^+^ 272, 286, and 300 from the reaction mixture with (*S*)-norcoclaurine (**1**) as substrate. **b** [M + H]^+^ 316, 330, and 344 from the reaction mixture with (*S*)-3′-hydroxy-*N*-methylcoclaurine (**6**) as substrate. **c** [M + H]^+^ 342 and 356 from the reaction mixture with (*S*)-tetrahydrocolumbamine (**9**) as substrate. **d** [M + H]^+^ 342 and 356 from the reaction mixture with tetrahydrojatrorrhizine (**11**) as substrate. **e** [M + H]^+^ 338 and 352 from the reaction mixture with columbamine (**12**) as substrate. **f** [M + H]^+^ 338 and 352 from the reaction mixture with jatrorrhizine (**14**) as substrate. **g** [M + H]^+^ 328, 342, and 356 from the reaction mixture with (*S*)-scoulerine (**15**) as substrate.

The substrates selected as representative of 1-BIAs were (*S*)-norcoclaurine (**1**) and (*S*)-3′-hydroxy-*N*-methylcoclaurine (**6**, 3′HNMC). CyOMT2, CyOMT5, and CyOMT7, which fall into the clade of OMTs involved in biosynthesis of BIAs, accepted (*S*)-norcoclaurine (**1**) as a substrate; while clustered with flavonoids and phenylpropanoids, CyOMT3 also used (*S*)-norcoclaurine (**1**) as a substrate (Fig. 4a). CyOMT2, CyOMT3, CyOMT5, and CyOMT7 catalyzed methylation at the C6 position of (*S*)-norcoclaurine (**1**) to produce (*S*)-coclaurine (**2**) based on their retention times and MS spectra compared with authentic standard compounds. CyOMT7 had the highest catalytic conversion at 90.5% (Table 1), with the major product (*S*)-coclaurine (**2**) and a trace amount of the byproduct norarmepavine (**4**). CyOMT5 catalyzed both C6 and C7 positions of (*S*)-norcoclaurine (**1**) to produce norarmepavine (**4**). CyOMT2 also catalyzed methylation at the C4′ position of (*S*)-norcoclaurine (**1**) to produce minor compounds 4′-methyl-norcoclaurine (**3**) and 4′-methyl-coclaurine (**5**), based on their ESI [+]-CID (collision-induced dissociation) spectra (Supplementary Data Fig. S2). This scheme was validated by *in vitro* assays of CyOMT5, 7 and 2 catalyzing (*S*)-coclaurine (**2**) to produce norarmepavine (**4**) and 4′-methyl-coclaurine (**5**), respectively (Supplementary Data Fig. S3, Fig. 4a). CyOMT2, CyOMT3, CyOMT4, CyOMT5, and CyOMT7 accepted (*S*)-3′-hydroxy-*N*-methylcoclaurine (**6**) as a substrate to catalyze methylation at C4′ and C7, producing (*S*)-reticuline (**7**) and peak **8**, respectively (Fig. 4b). CyOMT2 had the highest catalytic efficiency, with 78.4% substrate conversion [(*S*)-reticuline (**7**); Table 1], while CyOMT5 had the highest efficiency in C7 methylation, with 28.2% substrate conversion (peak **8**; Table 1). Although authentic standards for peak **8** were not available, comparison with the ESI [+]-CID spectrum of (*S*)-3′-hydroxy-*N*-methylcoclaurine (**6**) suggested that peak **8** is a previously unidentified product, 7-methyl-3′-hydroxy-*N*-methylcoclaurine (Supplementary Data Fig. S4).

**Table 1:** Catalytic promiscuity of CyOMT1–7. Values represent the percentage of product converted from substrate. OMTs with more than 20% conversion of substrates are highlighted in green and with conversion below 20% are shown in blue. The main products and catalytic positions are marked with arrows and corresponding product numbers

$\includegraphics{\bwartpath uhac152t1}$

Nd, not detected by TOF–MS.

The substrates selected as representative of tetrahydroprotoberberine alkaloids were (*S*)-tetrahydrocolumbamine (**9**, THCB), (*S*)-tetrahydrojatrorrihizine (THJ), and (*S*)-scoulerine (**15**). All seven CyOMTs accepted (*S*)-tetrahydrocolumbamine (**9**) as a substrate to catalyze methylation of C2 to produce (*S*)-tetrahydropalmatine (peak **10**, Fig. 4c); this was validated with authentic standard compounds. CyOMT5 was the most efficient in this reaction with a 75.4% conversion rate (Table 1). CyOMT2, CyOMT4, CyOMT5, and CyOMT7 accepted (*S*)-tetrahydrojatrorrihizine (THJ) as a substrate to catalyze methylation at the C3 position, producing (*S*)-tetrahydropalmatine (peak **10**, Fig. 4d). CyOMT5 had the highest catalytic conversion rate (43.5%), followed by CyOMT7 (34.2%), CyOMT4 (26.6%), and CyOMT2 (8.1%). The seven CyOMTs showed different catalytic properties between the C2 and C9 positions, as tested by the substrate (*S*)-scoulerine (peak **15**, Fig. 4g). All of the CyOMTs catalyzed methylation at the C2 position to produce peak **16**; furthermore, CyOMT2, CyOMT4, CyOMT5, and CyOMT7 catalyzed the product peak **16** to produce trace amounts of (*S*)-tetrahydropalmatine (**10**). For this reaction, CyOMT5 had the highest efficiency at the C2 position, with a 29.6% conversion rate (Table 1), and peak **16** was proposed to be tetrahydropalmatrubine based on the published MS spectra [5] (Supplementary Data Fig. S5). CyOMT6 efficiently catalyzed both 9- and 2-*O*-methylation of (*S*)-scoulerine (**15**) to produce (*S*)-tetrahydrocolumbamine (**9**) and (*S*)-tetrahydropalmatine (peak **10**, Fig. 4g).

The substrates selected as representative of quaternary protoberberine alkaloids were columbamine (**12**) and jatrorrhizine (**14**). CyOMT4, CyOMT5, CyOMT6, and CyOMT7 catalyzed methylation of columbamine (**12**) C2 to produce palmatine (**13**), as determined by the retention time and MS spectra compared with authentic standard compounds (Fig. 4e). CyOMT5 had the highest catalytic conversion, at 33.5% (Table 1), followed by CyOMT7 (24.6%), CyOMT4 (< 1%), and CyOMT6 (< 1%). CyOMT4, CyOMT5, and CyOMT7 additionally catalyzed methylation of jatrorrhizine (**14**) C3 to produce palmatine (**13**), but all had low conversion rates of no more than 10% (Fig. 4f).

In conclusion, seven CyOMTs were functionally tested for methylation activity using BIA intermediates with hydroxy sites. CyOMT2 (4′OMT) showed a preference for 1-BIAs and displayed the highest catalytic efficiency in 4′-*O*-methylations of (*S*)-3′-hydroxy-*N*-methylcoclaurine (**6**), and efficiently catalyzed 6-*O*-methylation of (*S*)-norcoclaurine (**1**), with minor byproducts of 4′-*O*-methylation (Fig. 4a and b). Both CyOMT5 (S2OMT) and CyOMT7 (6OMT) could accept three kinds of candidate compounds as substrates, with the C7 site of isoquinoline being the preferential site for CyOMT5; this produced the previously unknown product 7-methyl-3′-hydroxy-*N*-methyl-coclaurine (peak **8** in Fig. 4b). Site C6 of isoquinoline was the preferential site for CyOMT7. These OMTs could be employed as enzymes for producing diversified BIAs (Fig. 4a–f). CyOMT6 (S9OMT) performed two sequential methylations at the C9 and C2 positions of (*S*)-scoulerine (**15**) to produce the product (*S*)-tetrahydropalmatine (peak **10**, Fig. 4g). In addition, CyOMT1, CyOMT3, and CyOMT4 clustered with genes involved in phenylpropanoid biosynthesis. They could also accept some of the BIA pathway intermediates as a substrate, though with low catalytic efficiency. CyOMT4 catalyzed both the C2 and C3 positions of tetrahydroprotoberberine and quaternary protoberberine, and showed a high conversion rate for catalyzing methylation at the C2 position of tetrahydrojatrorrhizine (26.6%; Table 1, Fig. 4d). OMTs with >20% conversion of substrates are highlighted in green in Table 1. Collectively, the results show that these OMTs are promising candidates for use in synthetic biology applications to produce diversified BIAs.

### Modeling-based mutations of *Corydalis yanhusuo O*-methyltransferases revealed key residues related to enzyme promiscuity

**Figure 5:**
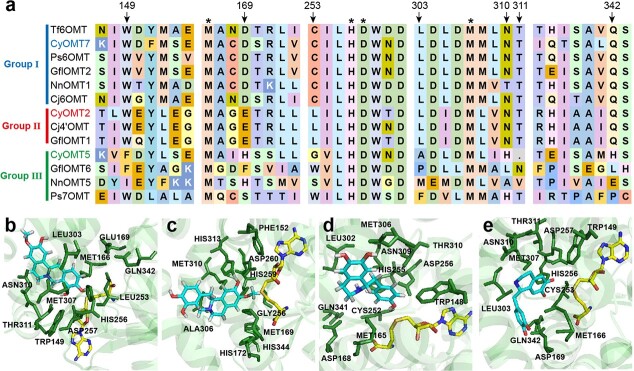
Homology modeling analysis of CyOMTs. **a** Multiple sequence alignment of previously characterized OMTs of three groups. Enzymes of Group I, which preferentially function as 6-*O*-methylation enzymes of 1-BIAs, are marked with a blue line. Enzymes of Group II, which preferentially function as 4′-*O*-methylation enzymes of 1-BIAs, are marked with a red line. Enzymes of Group III, which preferentially function as 7-*O*-methylation enzymes of *N*-methylated 1-BIAs, are marked with a green line. Asterisks indicate four highly conserved candidate amino acids (M166, H256, D257, and M307 in Tf6OMT), and the arrows point to the seven conserved residues (W149, D169, C253, L303, N310, T311, and Q342 in Tf6OMT). **b**–**d** Homology modeling of CyOMT2, CyOMT5, and CyOMT7. *S*-adenosy-*L*-methionine and substrate are marked in yellow and blue, respectively. **b** (*S*)-3′-hydroxy-*N*-methylcoclaurine (**6**) docked into CyOMT2. **c** (*S*)-3′-hydroxy-*N*-methylcoclaurine (**6**) docked into CyOMT5. **d** (*S*)-norcoclaurine (**1**) docked into CyOMT7. **e** Norlaudanosoline docked into Tf6OMT. The amino acid structures of the 11 conserved residues are labeled.

Biosynthesis of 1-BIAs is the central pathway in biosynthesis of BIAs (Fig. 1), and these are then catalyzed to produce various BIAs, including codeine, noscapine and so on. In order to identify key residues for the functional promiscuity of CyOMTs, we integrated molecular docking and sequence alignment analysis of 13 well-characterized OMTs but with different catalytic preference involved in the biosynthesis of 1-BIAs [21, 22, 24, 25, 29]. Although most of the reported OMTs are promiscuous, these 13 enzymes can be classified into three groups according to their substrate preferences. The first group (Group I, Fig. 5a), including CyOMT7, have been characterized to preferentially catalyze 6-*O*-methylation of 1-BIAs, which has been recognized as a key step involved in the biosynthesis of BIAs [31]. The second group (Group II, Fig. 5a), including CyOMT2, were more likely to catalyze 4′-*O*-methylation of 1-BIAs. The third group (Group III, Fig. 5a), including CyOMT5, preferred to catalyze 7-*O*-methylation of *N*-methylated 1-BIAs. As the crystal structure of Tf6OMT has been solved and the function of it has been well characterized, homology modeling of CyOMTs was simulated by SWISS-MODEL using Tf6OMT (PDB ID: 5ICE) (with 53.10–67.15% sequence identity) as a template [29]. The Global Model Quality Estimation value and related parameters showed good quality control of the models (Supplementary Data Table S6). Docking CyOMT2 with (*S*)-3′-hydroxy-*N*-methylcoclaurine (**6**), CyOMT5 with (*S*)-3′-hydroxy-*N*-methylcoclaurine (**6**), CyOMT7 with (*S*)-norcoclaurine (**1**), and Tf6OMT with norlaudanosoline revealed that 11 sites located in the substrate pocket were putatively related to enzyme function. Among these, four residues were conserved in the OMTs. Therefore, the other seven residues were selected as site-directed mutation targets for structure–function analysis (Fig. 5).

**Figure 6:**
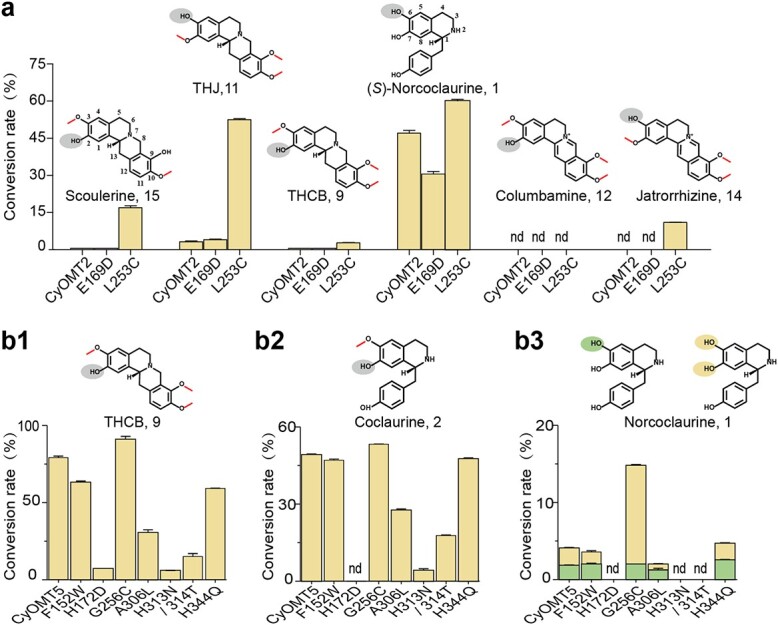
*In vitro* reactions of CyOMT mutants. *In vitro* reactions of CyOMT2 mutants on six BIAs (**a**) and CyOMT5 mutants on (*S*)-tetrahydrocolumbamine (**b1**), (*S*)-coclaurine (**b2**) and (*S*)-norcoclaurine (**b3**), separately. For each substrate, the sites of methylation are marked on the structure of the reaction substrate by elliptical pattern, which is color-coded to correspond to colors in the bar graph shown in panel **b3**. nd, not detected.

The mutants were purified and characterized as described previously (Supplementary Data Fig. S7). Sequence alignment showed that seven residues (W149, D169, C253, L303, N310, T311, and Q342 in Tf6OMT) were conserved in Group I enzymes (with the exception of N310 replaced by T in NnOMT1), but varied in Group II or Group III. Here, CyOMT2 and CyOMT5 were employed for mutation to analyze the roles of candidate residues in catalyzing 6 or 7-*O*-methylation. Five of the seven residues were conserved in Group I and Group II, except that the conserved sites D169 and C253 in Group I were replaced by E169 and L253 in Group 2. It was found that the L253C mutation of CyOMT2 greatly improved the 6-*O*-methylation activity and slightly improved the 7-*O*-methylation activity of isoquinoline; notably, it enhanced the conversion of tetrahydrojatrorrhizine (**11**) to (*S*)-tetrahydropalmatine (**10**) from ~5 to 52% (Fig. 6a). This site was also proved to be essential in catalyzing 6-*O*-methylation in CyOMT5 (Group III). Mutant CyOMT5_G256C seemed to slightly improve the 7-*O*-methylation activity by 1.1- to 1.2-fold but increase conversion of (*S*)-norcoclaurien (**1**) to (*S*)-norarmepavine (**4**) by nearly 9 times. This suggested that CyOMT5_G256C improves 6-*O*-methylation catalytic activity (Fig. 6b2 and b3). It was found that mutants/314T, H313N, A306L, and H172D of CyOMT5 exhibited a significant decrease in 7-*O*-methylation catalytic activity of isoquinoline of (*S*)-coclaurine (**2**) and (*S*)-tetrahydrocolumbamine (**9**) (Fig. 6b1 and b2), which indicated that these residues contribute to the function of 7-*O*-methylation of isoquinoline in CyOMT5. Furthermore, as phylogenetic analysis revealed that some 6OMTs and 7OMTs clustered together, these sites may separate the preference functionalities of 7OMTs from those of 6OMTs (Supplementary Data Fig. S6). The conserved key sites identified here provide a reference for the study of structure–function relationship of OMTs and could be proposed as marker sites for functional clarification of OMTs beyond phylogenetic analysis.

In order to further investigate the effect of key residue mutation on catalytic efficiency, the enzyme kinetic parameters of CyOMT2 and mutant CyOMT2_L253C, which has a positive effect on improving the yield of the products and expanding the selection of substrates, were determined. *K*_m_ and *k*_cat_ were calculated under the optimum reaction condition with pH of 7.5 and 45°C using norcoclaurine (**1**) as substrate, and with pH of 7.0 and 40°C using tetrahydrojatrorrhizine (**11**), scoulerine (**15**), and jatrorrhizine (**14**) as substrates (Supplementary Data Fig. S8). CyOMT2 and mutant L253C displayed a *K*_m_ value of 25.38 and 55.64 μM, respectively, for tetrahydrojatrorrhizine (**11**), while mutant L253C exhibited a significantly higher maximum reaction rate (*V*_max_) than CyOMT2. Catalytic efficiencies (*k*_cat_/*K*_m_) of mutant L253C were 9-fold higher than that of CyOMT2 for tetrahydrojatrorrhizine (**11**) as substrate and ~2-fold for norcoclaurine (**1**) as substrate. The methylation activities of mutant L253C using scoulerine (**15**) and jatrorrhizine (**14**) as substrate were relatively lower than those of norcoclaurine (**1**) (Supplementary Data Fig. S9, Table 2), which is consistent with the conversion results shown in Fig. 6a. The enzyme kinetic parameters showed that the mutant CyOMT2_L253C improved catalytic efficiency to the substrate above.

**Table 2:** Kinetic parameters of recombinant CyOMT2 and mutant CyOMT2_L253C for various substrates. Values represent the mean ± standard deviation of three independent measurements.

**Enzyme**	**Substrate**	***K***_**m**_ **(μM)**	***V***_**max**_ **(nmol min** ^**−1**^ **mg**^**−1**^**)**	***k***_**cat**_ **(s** ^**−1**^**)**	***k***_**cat**_ **/*K***_**m**_ **(M** ^**−1**^ **s**^**−1**^**)**
CyOMT2	Norcoclaurine, **1**	31.00 ± 5.60	10.63 ± 0.63	0.0099	318.56
	tetrahydrojatrorrhizine, **11**	25.38 ± 3.09	0.36 ± 0.01	0.0003	13.28
CyOMT2_L253C	Norcoclaurine, **1**	19.43 ± 3.39	11.19 ± 0.54	0.0104	535.02
	tetrahydrojatrorrhizine, **11**	55.64 ± 5.21	7.73 ± 0.20	0.0072	129.11
	Scoulerine, **15**	12.24 ± 1.72	0.74 ± 0.02	0.0007	56.13
	Jatrorrhizine, **14**	34.83 ± 3.70	0.57 ± 0.01	0.0005	15.18

### Co-expression of *O*-methyltransferases with accumulation of benzylisoquinoline alkaloids in *Corydalis yanhusuo*

**Figure 7:**
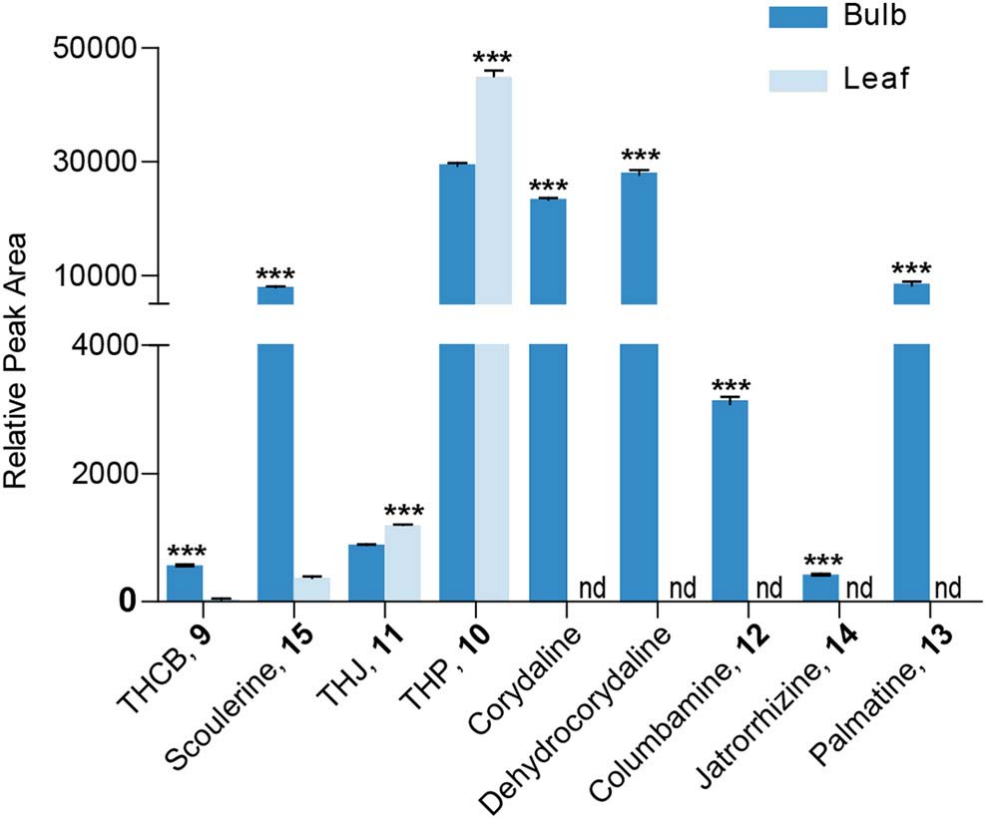
Relative content of alkaloids in leaf and bulb tissues of *C. yanhusuo*. Levels of tetrahydroprotoberberines, including scoulerine, tetrahydrocolumbamine, and corydaline, were significantly higher in bulb tissue, while tetrahydropalmatine and tetrahydrojatrorrhizine accumulated at similar levels in each tissue. All tested quaternary protoberberines, including dehydrocorydaline, columbamine, jatrorrhizine, and palmatine, were significantly higher in the bulb tissue. Error bars show standard error from three biological replicates. ^***^P < .001, unpaired *t*-test (*n* = 3). nd, not detected.

To further analyze the relevance of OMTs to BIA biosynthesis in *C. yanhusuo*, accumulation of biosynthetic pathway intermediates was compared between different tissues. Intermediate tetrahydroprotoberberines, including scoulerine (**15**), tetrahydrocolumbamine (**9**), and corydaline, were more abundant in the bulb, while tetrahydropalmatine (**10**) and tetrahydrojatrorrhizine (**11**) accumulated at similar levels in the bulb and leaf. All tested quaternary protoberberines, including dehydrocorydaline, columbamine (**12**), jatrorrhizine (**14)**, and palmatine (**13**), were significantly higher in the bulb tissue. However, intermediate 1-BIAs, including norcoclaurine (**1**), 3′-hydroxy-*N*-methylcoclaurine (**6**), and coclaurine (**2**), were not detected in either tissue (Fig. 7).

**Figure 8:**
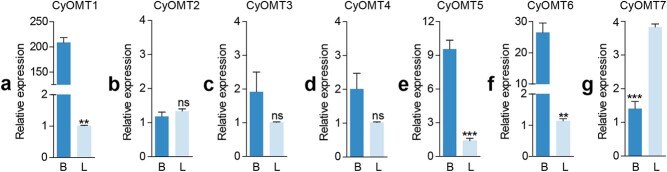
Relative gene expression of CyOMTs in *C. yanhusuo* leaf (L) and bulb (B) tissue. Expression of CyOMT2 and CyOMT7 was lower in bulb tissue than in leaf tissue; all other genes showed much higher expression in bulb tissue. Error bars show standard error from three independent replicates. Error bars show standard error from three independent replicates. ^**^P < .01; ^***^P < .001; ns, no significant difference; unpaired *t*-test (*n* = 3).

CyOMT5 and CyOMT6 expression was most abundant in the bulb, accumulating at 7- and 23-fold higher levels, respectively, compared with the leaf (Fig. 8). This is consistent with the accumulation pattern of the substrates and products of CyOMT5 and CyOMT6. Therefore, both gene expression and product accumulation support the idea that these two genes might be involved in biosynthesis of (*S*)-tetrahydrocolumbamine (**9**), (*S*)-tetrahydropalmatine (**10**), columbamine (**12**), and palmatine (**13**). Furthermore, the gene expression of CyOMT2 and CyOMT7 was higher in the leaf, especially CyOMT7, with leaf tissue accumulating 2.7-fold higher levels compared with the bulb (Fig. 8). Considering the abundant accumulation of (*S*)-tetrahydropalmatine (**10**) in leaf tissue (Fig. 7) and that CyOMT2 and CyOMT7 had the most catalytic activity with 1-BIA substrates out of all seven tested CyOMTs (Table 1), we propose that CyOMT2 and CyOMT7 play an important role upstream of the BIA biosynthetic pathway. They provide a basis for metabolic engineering of BIAs *in planta*.

## Discussion

Understanding of BIA metabolic pathways in plants has advanced rapidly over the past several decades. OMTs have an assignable influence in skeleton formation, structural diversity, and downstream enzymatic processing in BIA biosynthesis pathways. Here, seven OMTs from *C. yanhusuo* (CyOMTs) were isolated and functionally analyzed using seven compounds to represent three main types of BIAs in *C. yanhusuo*. By enzyme promiscuity analysis, it was speculated that promiscuity of OMTs plays an essential role in producing diversified BIAs *in planta*. Most of the substrates and catalytic products could be detected in *C. yanhusuo*, though some of them had a relatively low content, which might have resulted from the different affinity of OMTs for different substrates. The roles of these OMTs in producing chemical diversity of BIAs were further characterized, including the C6, C7, and C4′ positions of 1-BIAs, and the C2, C3, and C9 positions of tetrahydroprotoberberines and quaternary protoberberines.

Specifically, we found that CyOMT5 (as well as CyOMT3, 4, and 7) could employ 3′-hydroxy-*N*-methylcoclaurine as substrate to produce a new 7-*O*-methylated product (**8**). Moreover, CyOMT5 showed efficient methylation activity at specific sites (sites C6 and C7 of isoquinoline) in three types of BIA, indicating a potential for the development of this enzyme into a broadly useful tool for methyltransferase reactions. In addition, CyOMT3 and CyOMT6 catalyze the 2-*O*-methylation of (*S*)-tetrahydrocolumbamine (**9**), but not 3-*O*-methylation of tetrahydrojatrorrhizine (**11**). The selectivity between position C2 and C3 has not previously been reported, and may be helpful in future metabolic engineering studies related to the formation of tetrahydrojatrorrhizine (**11**), jatrorrhizine (**14**), or similar compounds. Scoulerine 9-*O*-methyltransferase (S9OMT), catalyzing the conversion of (*S*)-scoulerine (**15**) to (*S*)-tetrahydrocolumbamine (**9**), has been recognized as a committed step in noscapine and berberine biosynthesis pathways [5, 32]. Recently, the crystal structures of S9OMTs from *P. somniferum* [32] and *T. flavum* [5] were published, which is expected to guide protein engineering for targeted production of BIAs. Here, we identified an S9OMT (CyOMT6) catalyzing both 9-*O*- and 2-*O*- of scoulerine (**15**). Such OMTs offer valuable natural variation for structure–functional analysis.

Although catalytic promiscuity of OMTs is common in the biosynthesis of BIAs, decoration of the benzylisoquinoline backbone, including methylation of free hydroxyl groups of other non-catalytic sites, or *N*-methylation generating quaternary amines, greatly affects the efficiency of OMTs. For example, CyOMT5 efficiently catalyzes (*S*)-coclaurine (**2**) with 49% conversion, while for (*S*)-norcoclaurine (**1**) conversion was 4%, despite the only difference between the two compounds being a 6-*O*-methylation. CyOMT4, CyOMT5, CyOMT6, and CyOMT7 catalyzed methylation of tetrahydroprotoberberines and quaternary protoberberines, but they showed different catalytic efficiency between these two types of substrate. This may be related to *N*-methylation, which influences overall hydrophobicity, steric bulk, and conformations of the molecule [19]. This catalytic difference between tertiary and quaternary amine-type substrates has also been found in previous studies. For example, *P. somniferum* norreticuline 7-*O*-methyltransferase (PsN7OMT) is able to accept norreticuline but not reticuline as a substrate, but all tested quaternary amine-type substrates compounds were not converted by PsN7OMT in a previous study [33]. Thus, abundant substrate levels with minor changes in the backbone are essential for accurate functional characterization of OMTs. In summary, catalytic promiscuity of OMTs allows comprehensive study of the structural basis underlying functional preference, which is expected to aid in the artificial design of proteins and structure-guidedengineering [19].

Phylogenetic analysis of 75 characterized OMTs showed that, in general, enzymes with the same function clustered together, which provides a reference for functional characterization. For example, CyOMT2, CyOMT5, CyOMT6, and CyOMT7 cluster with genes involved in the BIA pathway and could efficiently catalyze BIA substrates and with abundant catalytic sites, while CyOMT1, CyOMT3, and CyOMT4 were found to cluster with genes involved in flavonoid, phenylpropanoid or benzenoid phenolic pathways, and showed relatively weak catalytic efficiency with BIAs. Given the interest in promiscuity of OMTs, we conducted docking analysis to characterize 13 OMTs catalyzing the decoration of different methylation positions in 1-BIAs as an example for structure–function analysis. This analysis showed that seven residues in the 1-BIA-binding domain [29] were conserved among one group of the 13 OMTs but not the other two, consistent with observed differences in catalytic function. Both CyOMT2_L253C and CyOMT5_G256C showed the characteristics of strengthening the function of 6-*O*-methylations of 1-BIAs, especially for CyOMT2, greatly improving methylation of tetrahydrojatrorrhizine (**11**) from ~5 to 52%. These results provide experimental support for the criticality of Cys at site 253 in CyOMT2 (253 in Tf6OMT), a residue in direct contact with the 6-hydroxyl group of substrates shown in the crystal structure of Tf6OMT. Moreover, the mutation of residues 313N and 314T in CyOMT5 (310N and 311T in Tf6OMT), which stabilize the A and C ring positions of 1-BIA, also had a great impact on catalytic activity [29]. These results encourage further examination for catalytic promiscuity of OMTs by such modeling (tertiary structure)- and sequence alignment (primary structure)-based mutations.

In summary, here we isolated seven OMTs from the Chinese medicinal herb *C. yanhusuo* based on the screening principle of high gene expression and characterized their activity using seven potential BIA substrates. Highly efficient OMTs responsible for C6, C7, and C4′ methylation of 1-BIA and C2, C3, and C9 methylation of tetrahydroprotoberberines or quaternary protoberberines were identified. Furthermore, 13 OMTs were employed as examples for structure–function analysis based on phylogenetic analysis and molecular docking, leading to the identification of several residues related to promiscuity of *O*-methylations at the 6- and 7-positions of 1-BIAs; specifically, mutant CyOMT2_L253C significantly increases catalytic efficiency and expands substrate scope in comparison with CyOMT2. This work gives detailed insight into OMTs in the formation of BIA diversity from *C. yanhusuo* and provides genetic elements for synthetic production of BIAs, which will be valuable for their production by synthetic biology. It also paves the way for studying the molecular basis related to the catalytic promiscuity of OMTs involved in BIA biosynthesis. Further studies on enzyme engineering and the production of BIAs by metabolic engineering will be conducted.

## Materials and methods

### Plant materials and chemicals

*C. yanhusuo* was acquired from Jiangxi Cheersome Agriculture Development Co., Ltd in Heng Feng county, Jiangxi Province, China, in April 2019. Plants were collected and divided into bulb and leaf tissues. Both tissues were flash-frozen in liquid nitrogen and stored at −80°C.

Authentic (*S*)-norcoclaurine (**1**), (*S*)-3′-hydroxy-*N*-methylcoclaurine (**6**), (*S*)-reticuline (**7**), and (*S*)-scoulerine (**15**) were obtained from Toronto Research Chemicals Inc. (Toronto, Canada). (*S*)-tetrahydrocolumbamine (**9**), (*S*)-tetrahydrojatrorrhizine (**11**), jatrorrhizine (**14**), columbamine (**12**), palmatine (**13**), norarmepavine (**4**), (*S*)-tetrahydropalmatine (**10**), (*S*)-coclaurine (**2**), corydaline, and dehydrocorydaline alkaloid were obtained from Shanghai Yuanye Bio-Technology Co., Ltd. *S*-Adenosylmethionine was purchased from Sigma–Aldrich.

### RNA extraction

Total RNA was extracted from leaf and bulb tissues of *C. yanhusuo* using an RNA isolation kit (HuaYueYang Biotechnology, Beijing, China). RNA concentration, purity, and integrity were measured using a Qubit^®^ RNA Assay Kit in the Qubit^®^ 2.0 Fluorometer (Life Technologies, CA, USA), a NanoPhotometer^®^ spectrophotometer (CA, USA), and the Agilent Bioanalyzer 2100 system (CA, USA), respectively.

### Transcriptome sequencing, *de novo* assembly and annotation

Transcriptome sequencing was conducted by Novogene and sequencing libraries were generated using NEBNext^®^ Ultra™ RNA Library Prep Kit for Illumina^®^. Clustering of samples was performed on a cBot Cluster Generation System using TruSeq PE Cluster Kit v3-cBot-HS. The library preparations were made on an Illumina Hiseq platform and paired-end reads were generated. The reads were *de novo* assembled and estimated using the Trinity pipeline. Gene functions were annotated based on data from the NCBI [34] Nr and Nt databases and from Pfam [35], the COG/KOG database [36], Swiss-Prot [37], the KEGG database [38], and the GO database [39].

### Cloning, sequence alignment, and phylogenetic analysis of *CyOMT* genes

First-strand cDNA synthesis was performed on 1 μg of RNA per sample using the PrimeScript RT Reagent Kit with DNA Eraser (Takara, Tokyo, Japan). Open reading frames of CyOMTs were amplified from cDNA using PrimeSTAR HS DNA Polymerase (Takara) with gene-specific primers with a vector overlapping sequence (Supplementary Data Table S2) and cloned into the vector pET32a with a Seamless Cloning Kit (TransGen Biotech, Beijing, China). Recombinant plasmids were transformed into *E. coli* Trans T1 (TransGen) and verified by both PCR (using 2× EasyTaq PCR SuperMix; TransGen) and sequencing. CyOMT sequence alignment was visualized with Jalview [40]. The previously identified 68 OMTs from the literature (Supplementary Data Table S4) together with the seven successfully cloned OMTs from *C. yanhusuo* produced a phylogenetic tree using MEGA 7 [41]. We used ClustalW [42] for amino acid alignment and the neighbor-joining method for phylogenetic analysis with 1000 bootstrap replicates. The *p*-distance model was used to calculate the genetic distance, and the gap/missing data treatment used was partial deletion, with 50% site coverage cutoff.

### Recombinant protein purification

Recombinant plasmids were expressed in *E. coli* BL21 (DE3) (TransGen) and coated in Luria agar containing 100 μg/mL ampicillin overnight at 37°C. Single colonies were inoculated into LB medium (5 mL per sample, containing 100 μg/mL ampicillin), and cultures were shaken at 37°C and 200 rpm for 5 hours. The 5-mL cultures were then transferred to 500 mL LB medium (with 100 μg/mL ampicillin), and incubated with shaking until the OD_600_ reached 0.6–0.8. The temperature was dropped to 17°C for 30 minutes prior to induction with a final concentration of 0.5 mmol/L isopropyl-β-d-thiogalactopyranoside (IPTG). Cultures were then incubated at 17°C with shaking at 200 rpm for 16 hours. The cells were isolated and enriched by centrifugation (8000 g for 10 minutes at 4°C) and resuspended in 50 mL protein extraction buffer (100 mmol/L Tris–HCl, 300 mmol/L NaCl, pH 7.4). Phenylmethanesulfonyl fluoride was added at the concentration of 1 mmol/L and the cells were then disrupted by ultrasonication (Branson Digital Sonifier, USA; 10% amplitude, 30 minutes, 3 seconds on, 3 seconds off) to obtain crude protein. After centrifugation at 13 000 g for 15 minutes, 1 mL Ni agarose resin (CoWin, Jiangsu, China) was added to the supernatant. Samples were then washed with 20 mL of protein extraction buffer followed by 30 mL of solution binding buffer (20 mmol/L imidazole). Finally, the target protein was eluted with 3 mL of elution buffer (250 mmol/L imidazole). The collected protein components were concentrated and purified by repeated ultrafiltration with an Amicon Ultra 15 K column (EMD Millipore, Burlington, MA, USA) in 100 mmol/L Tris–HCl (pH 7.4). Protein concentration and purity were tested with a Bradford kit (TransGen) and polyacrylamide gel electrophoresis (10% gel). The purified protein was stored at −80°C in 30% glycerol (v/v).

### Functional characterization of the recombinant *Corydalis yanhusuo O*-methyltransferases and mutants

OMT activity was tested in a total of 200 μL assay buffer (100 mmol/L Tris–HCl, pH 7.5), including 100 μM substrate, 500 μM AdoMet, and 50 μg purified protein. Protein extracted from *E. coli* containing the pET32a vector was used as the negative control. Reactions were vibrated at 37°C and 150 rpm for 3 hours and terminated with 400 μL methanol. Terminated reaction samples were passed through a 0.22-μm filter; filtrate was quantitatively analyzed by UPLC. Some products without available authentic standards cannot be quantified and contents of these products were calculated by a semiquantitative method, i.e. the product was quantified by the linear relationship between concentration and absorbance value of the corresponding substrate. Products detected by UPLC at the absorption wavelength of 284 nm were used to calculate conversion with the following formula:

Specific site conversion (%) = 100 × corresponding product content/(all product content + substrate content).

To determine the optimal pH, CyOMT2_L253C assays were conducted in 50 mM citric acid–sodium citrate (pH 5.0–6.0), 50 mM NaH_2_PO_4_-Na_2_HPO_4_ (pH 6.0–7.5), 100 mM Tris–HCl (pH 7.5–9.0), or 100 mM Na_2_CO_3_-NaHCO_3_ (pH 9.0–10.0), using 100 μM norcoclaurine (**1**), tetrahydrojatrorrhizine (**11**), scoulerine (**15**) or jatrorrhizine (**14**) as substrate, with 200 μM SAM and 25 μg purified recombinant protein in a reaction volume of 100 μL. Assays were incubated at 37°C and 150 rpm for 3 hours and terminated with 200 μL methanol. Temperature optima were determined under the same conditions but in 100 mM Tris–HCl (pH 7.5) and incubation temperatures between 25 and 55°C were used. *K*_m_ and *k*_cat_ were calculated under the optimum reaction condition with pH of 7.5 (50 mM NaH_**2**_PO_**4**_-Na_**2**_HPO_**4**_) and 45°C using norcoclaurine (**1**) as substrate, and with pH of 7.0 (50 mM NaH_**2**_PO_**4**_-Na_**2**_HPO_**4**_) and 40°C using tetrahydrojatrorrhizine (**11**), scoulerine (**15**), and jatrorrhizine (**14**) as substrates, with alkaloid concentrations from 0 to 1200 μM and SAM concentration of 200 μM in a reaction volume of 100 μL. Reactions were incubated with shaking at 150 rpm for 30 minutes and ended with 200 μL methanol. Finally, the kinetic parameters were analyzed based on the Michaelis–Menten equation.

### UPLC–QTOF–MS analysis

Catalytic products were analyzed by UPLC–QTOF–MS, coupling a Waters Acquity UPLC-I-Class instrument and a Waters Xevo G2-S QTof. A reversed-phase chromatographic T3 column (2.1 mm × 100 mm, 2.7 μm particle size) was used with flow rate 0.5 mL/minute at 40°C. Analytes were eluted using a mobile phase gradient of solvent A, comprising 0.1% (v/v) formic acid and 99.9% (v/v) water, and solvent B, comprising 0.1% (v/v) formic acid and 99.9% (v/v) acetonitrile. Alkaloids were eluted under the following conditions: 5%–30% solvent B from 0.0 to 6.0 minutes, 30–60% solvent B from 6.0 to 12.0 minutes, 60–90% solvent B from 12.0 to 13.5 minutes, 90–5% solvent B from 13.5 to 15.0 minutes, and 5% solvent B from 15 to 17 minutes. For each sample, 1 μL of filtrate was injected into the system. The following QTOF–MS parameters were used: mass spectrometer equipped with electrospray ionization (ESI) mode in positive ion mode; scan range, 50–1500 Da; scan time, 0.1 s; ramp collision energy, 30–50 V. Data analysis was performed using MassLynx (Waters Technologies).

### Measurement of alkaloid content

UPLC–QTOF–MS was used to determine the relative content of alkaloids in *C. yanhusuo*. Tissues frozen at −80°C were ground into fine powder with a ball mill then freeze-dried in lyophilizer FDU1110 (Eyela, Tokyo, Japan). The extraction method was as follows: 50.0 mg of dried plant tissue was accurately weighed in a 2-mL tube and 1 mL of extracting solution (80% methanol, 20% water [v/v]) was added. Samples were incubated for 5 hours and extracted by ultrasonication (Scientz, Zhejiang, China) for 30 minutes then centrifuged at 13 000 g for 15 minutes. Supernatant (100 μL) was transferred to a 1.5-mL tube and diluted 1:9, and the same operation was repeated for a final concentration of 1% due to the high content of alkaloids in *C. yanhusuo* and because the tailing phenomenon of alkaloids seriously affects chromatographic separation. Samples were passed through a 0.22 μm filter and tested with the same parameters described above otherwise specified. Alkaloids were eluted under the following conditions: 10–25% solvent B from 0.0 to 15.0 minutes, 25–50% solvent B from 15.0 to 20.0 minutes, 50–95% solvent B from 20.0 to 24.0 minutes, 95% solvent B from 24.0 to 25.0 minutes, 95–10% solvent B from 25.0 to 25.5 minutes, and 10% solvent B from 25.5 to 28.0 minutes. For each tissue, three biological replicates were measured. Exact masses, retention times, and CID spectra of available authentic standards were used for alkaloid identification.

### Gene expression analysis (quantitative real-time PCR analysis)

RNA was extracted as described above, then reverse-transcribed by PrimeScript RT reagent Kit with DNA Eraser (Takara); each 20-μL reaction contained 1 μg RNA. The quantitative real-time PCR (qPCR) was performed in the LightCycler 480 II Real-Time PCR detection system. Primer sequences are shown in Supplementary Data Table S3. Ubiquitin C was used as the internal control and relative transcript abundance was calculated by the 2^-ΔΔ^Ct method [43]. Three independent replicates were tested per sample.

### Molecular docking and site-directed mutagenesis

Protein structure homology was modeled with SWISS-MODEL [44]. Docking calculations were performed by AutoDock4 [45]. The crystal structure of (*S*)-norcoclaurine 6-*O*-methyltransferase with *S*-adenosylhomocysteine and norlaudanosoline was loaded from RCSB [46] (https://www.rcsb.org/), and the catalytic region of the substrate refers to this complex structure in the process of docking.

The mutations of the CyOMTs were cloned by PCR amplification with a Fast Mutagenesis System (TransGen). Using methylated plasmid as template, primers were designed by the partial overlap or full overlap method (shown in Supplementary Data Table S3) and amplified by 2× TransStart FastPfu Fly PCR SuperMix. After digesting the methylated plasmid with the DMT restriction enzyme, the amplified product was transformed into competent cells that could degrade the methylated plasmid. The previously described method in the section of Recombinant protein purification was used for recombinant protein purification and functional characterization.

## Acknowledgements

This work was supported by the National Key R&D Program of China (2020YFA0908000), the National Natural Science Foundation of China (31961133007, 82011530137, 81822046, 81891010, 82003904), a key project at central government level (the ability to establish sustainable use of valuable Chinese medicine resources; 2060302, China), the Fundamental Research Funds for the Central Public Welfare Research Institutes (ZZ13-YQ-083), the Innovation Team and Talents Cultivation Program of National Administration of Traditional Chinese Medicine (No. ZYYCXTD-D-202005) and a Scientific and Technological Innovation Project of China Academy of Chinese Medical Sciences (CI2021A04110). We acknowledge support from Vetenskapsrådet and Stiftelsen för Internationalisering av Högre Utbildning och Forskning.

## Author contributions

J.B.: conceptualization, methodology, and original draft preparation. X.Z.: molecular docking and *in vitro* experiment on directed mutagenesis. Q.L., Y.M., and Z.H.: methodology and molecular modeling. J.Y.: chemical analysis. X.L., Y.C., X. J., and R.W.: guidance of *in vitro* expression experiment. T.C.: bioinformatics analysis. C.L., S.L., and Y.K.: chemical analysis. G.C., J.T., and L.Y.: data analysis, reviewing. J.G.: investigation, validation, writing, reviewing, supervision, and editing. L.H.: funding acquisition, project administration, and resources.

## Data availability

The transcriptome reported in this paper has been deposited in the China National Center for Bioinformation under accession number PRJCA CRA005108, which is publicly accessible for all researchers at http://bigd.big.ac.cn/gsa.

## Conflict of interest

The authors declare that they have no conflict of interest, in accordance with the journal policy.

## Supplementary data

Supplementary data is available at *Horticulture Research* online.

## Supplementary Material

Web_Material_uhac152
