# Supplementary material for: Catalytic promiscuity of O-methyltransferases from Corydalis yanhusuo leading to the structural diversity of benzylisoquinoline alkaloids
Source: Hortic Res. 2022 Jul 6;9:uhac152. doi: 10.1093/hr/uhac152 (PMC9510826; doi:10.1093/hr/uhac152)
Supplement: Web_Material_uhac152 [file web_material_uhac152.docx]

**SUPPORTING INFORMATION for**

**Catalytic promiscuity of *O*-methyltransferases from *Corydalis yanhusuo* leading to the structural diversity of benzylisoquinoline alkaloids**

Junling Bu^a,1^, Xiuhua Zhang^a,1^, Qishuang Li^a^, Ying Ma^a^, Zhimin Hu^a^, Jian Yang^a^, Xiuyu Liu^b^, Ruishan Wang^a^, Xiang Jiao^c^, Tong Chen^a^, Changjiangsheng Lai^a^, Guanghong Cui^a^, Jinfu Tang^a^, Yu Kong^d^, Lei Yang^d^, Sheng Lin^e^, Yun Chen^c^, Juan Guo^a*^ and Luqi Huang^a*^

^a^State Key Laboratory Breeding Base of Dao-di Herbs, National Resource Center for Chinese Materia Medica, China Academy of Chinese Medical Sciences, No. 16 South Side Street, Dongzhimen, Beijing 100700, China

^b^School of Pharmacy, Henan University of Chinese Medicine, No. 156 Jinshuidong Road, Zhengzhou 450008, China

^c^Department of Biology and Biological Engineering, Chalmers University of Technology, Kemivägen 10, SE41296, Gothenburg, Sweden

^d^Shanghai Key Laboratory of Plant Functional Genomics and Resources, Shanghai Chenshan Botanical Garden, Shanghai, 201602, China

^e^Key Laboratory of Chinese Internal Medicine of Ministry of Education and Beijing, Dongzhimen Hospital, Beijing University of Chinese Medicine, Beijing, 100700, China

^*^For correspondence: guojuan@wbgcas.cn (Juan Guo), huanglq@cacms.cn (Luqi Huang)

**Fig. S1** Molecular cloning and purification of recombinant protein. (a) Agarose gel electrophoresis analysis of CyOMT1-7 cloning. Lane M: DNA marker; Lanes 1-7: cloned open reading frames of CyOMT1-7, respectively. (b) SDS-PAGE analysis for purified CyOMTs. Lane M: molecular weight marker; Lane P: Crude protein extract from *E. coli* containing the empty vector; Lanes1-7: purified enzymes of CyOMT1-7, respectively.


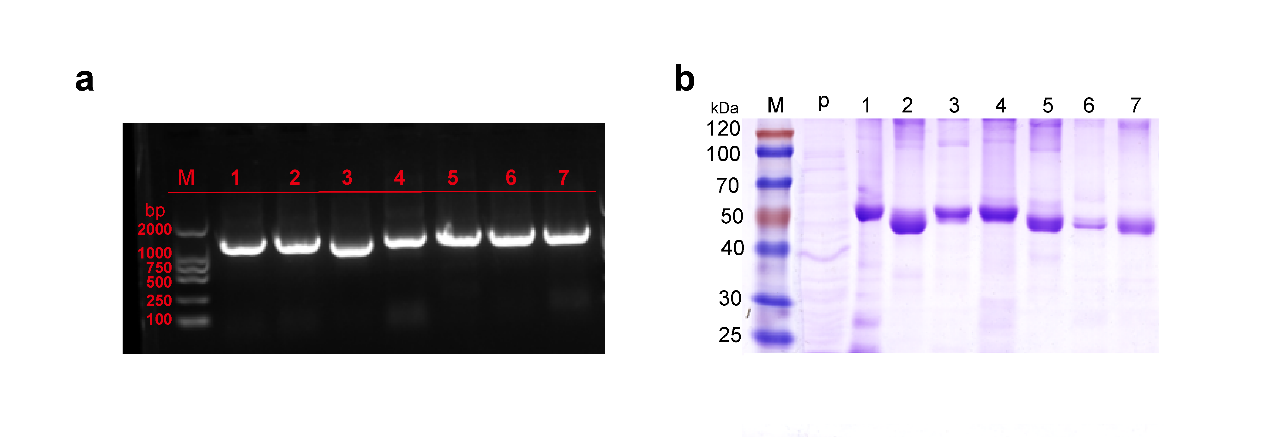


**Fig. S2** Identification of the *in vitro* reaction products of peaks **3** and **5**. Collision-induced dissociation (CID) analysis and MS/MS fragmentation spectrum of peaks **3** and **5** were shown.


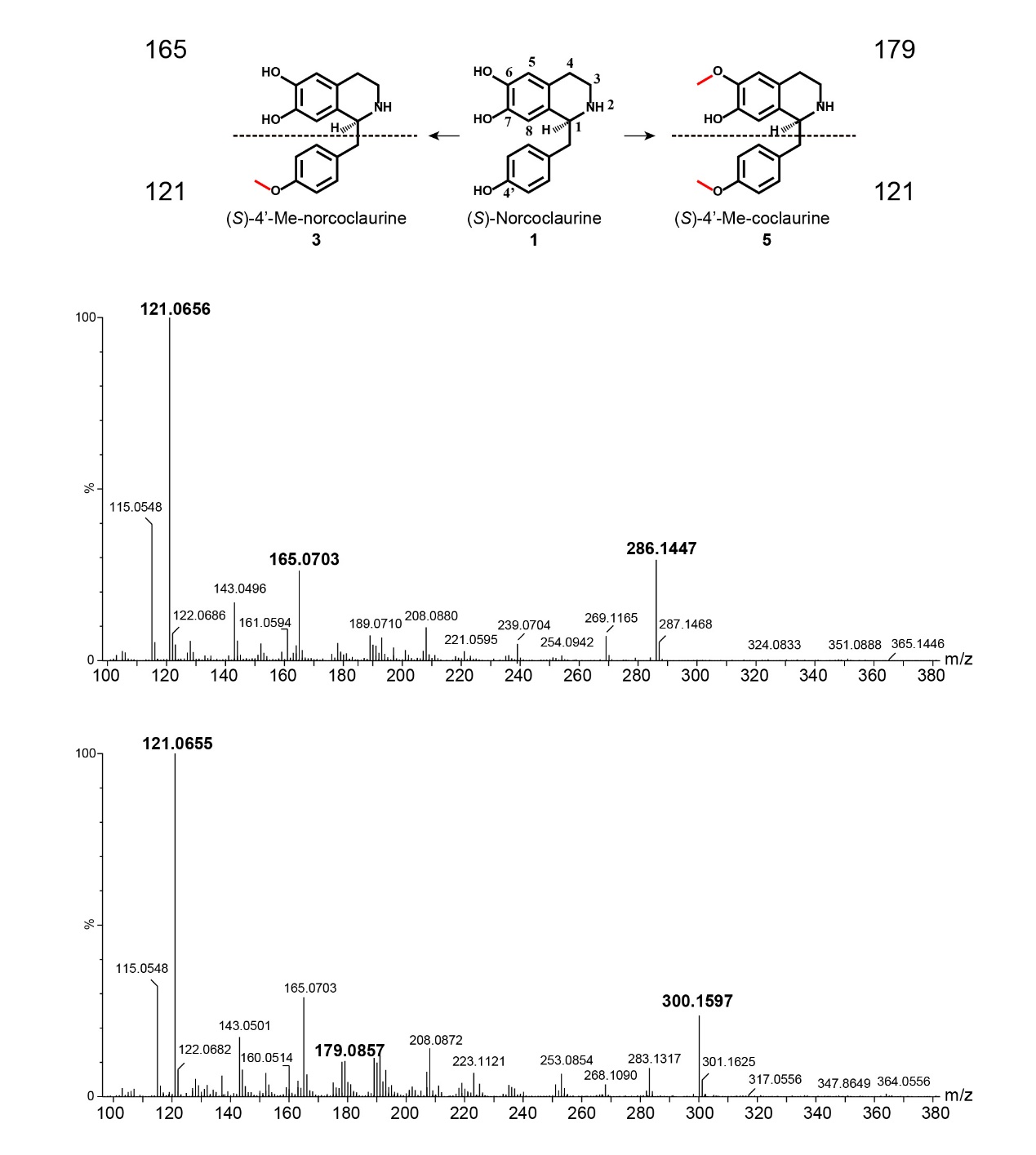


**Fig. S3** Extracted ion chromatogram (EIC) showing the *O*-methylation activity of CyOMT5, CyOMT7 (a) and CyOMT2 (b) with (*S*)-coclaurine as substrate. Protein extracted from *E. coli* containing the empty vector pET32a was used as control. CyOMT5 and CyOMT7 catalyzing methylation at the C7 position of (*S*)- coclaurine (**2**) to produce (*S*)-norarmepavine (**4**) based on their retention time and MS spectra compared with authentic standard compounds. CyOMT2 catalyzing methylation at the C4’ position of (*S*)-coclaurine to produce 4’-methyl-coclaurine (**5**), based on their ESI [+]-CID spectra showed in Fig. S2.


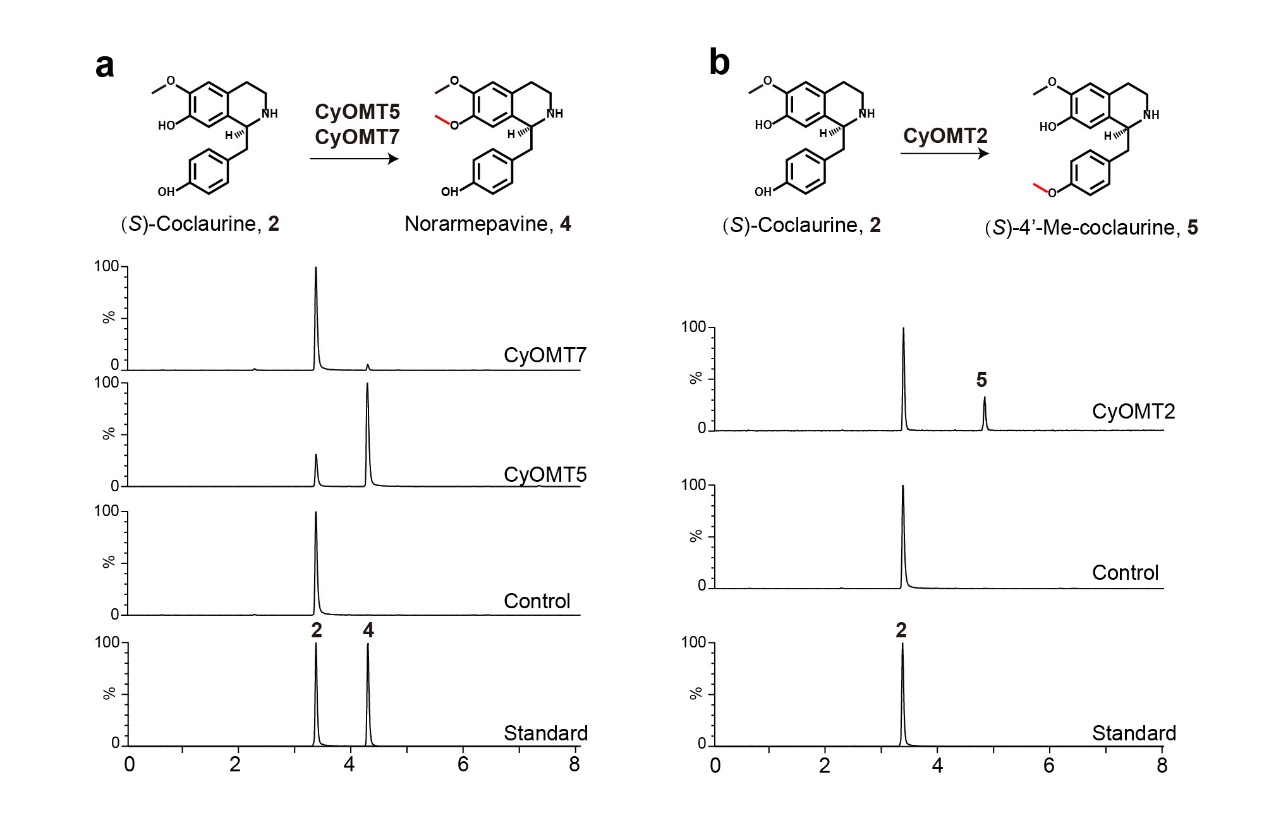


**Fig. S4** Identification of the *in vitro* reaction products of peak **8**. Collision-induced dissociation (CID) analysis and MS/MS fragmentation spectrum of peak8 and (*S*)-3’-hydroxy-*N*-methylcoclaurine (**6**) were shown.


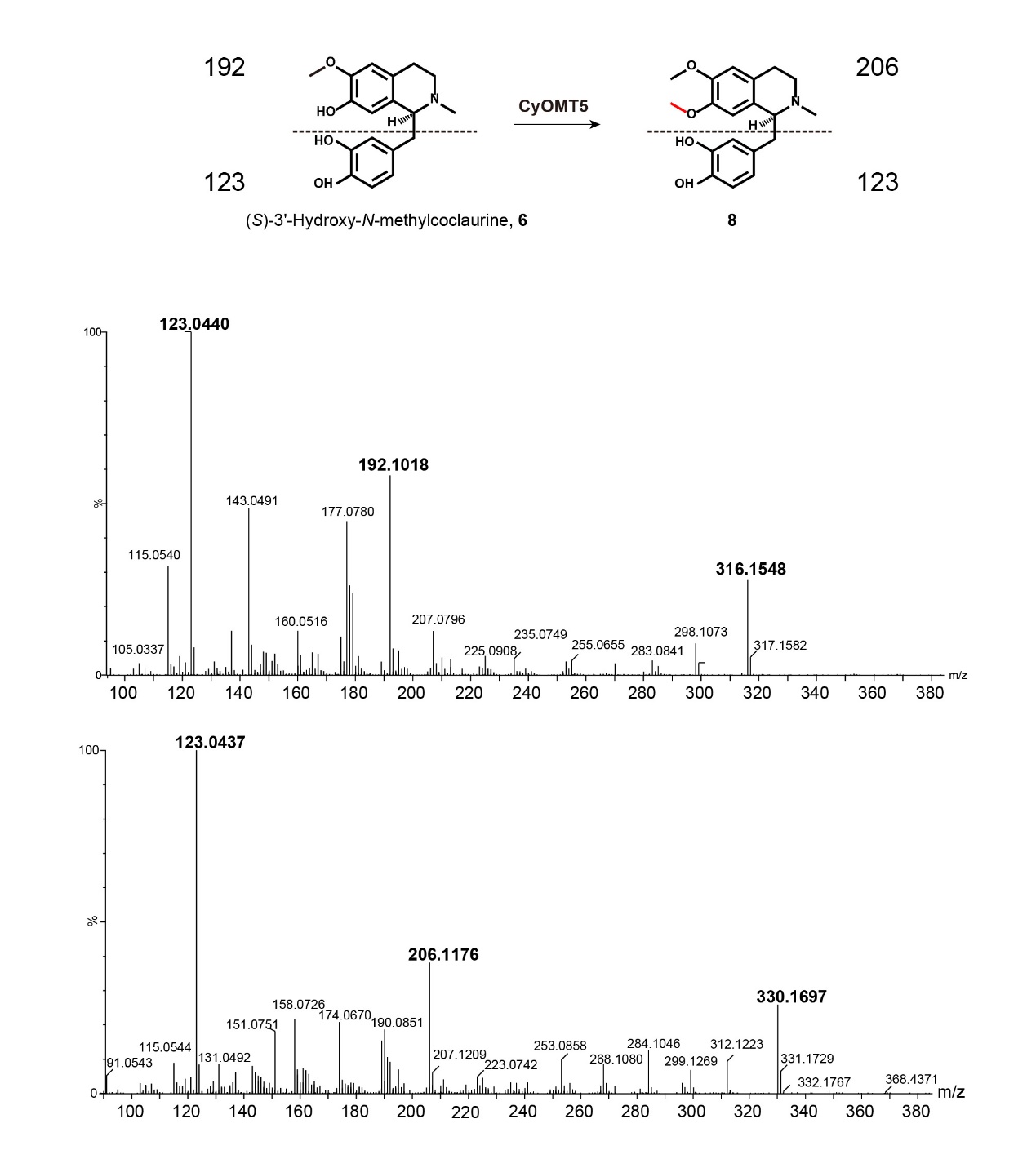


**Fig. S5** Identification of the *in vitro* reaction products of peak **16**. Collision-induced dissociation (CID) analysis and MS/MS fragmentation spectrum of peak **16** were shown. Peak **16** was proposed to be tetrahydropalmatrubine based on the published MS spectra^1^.


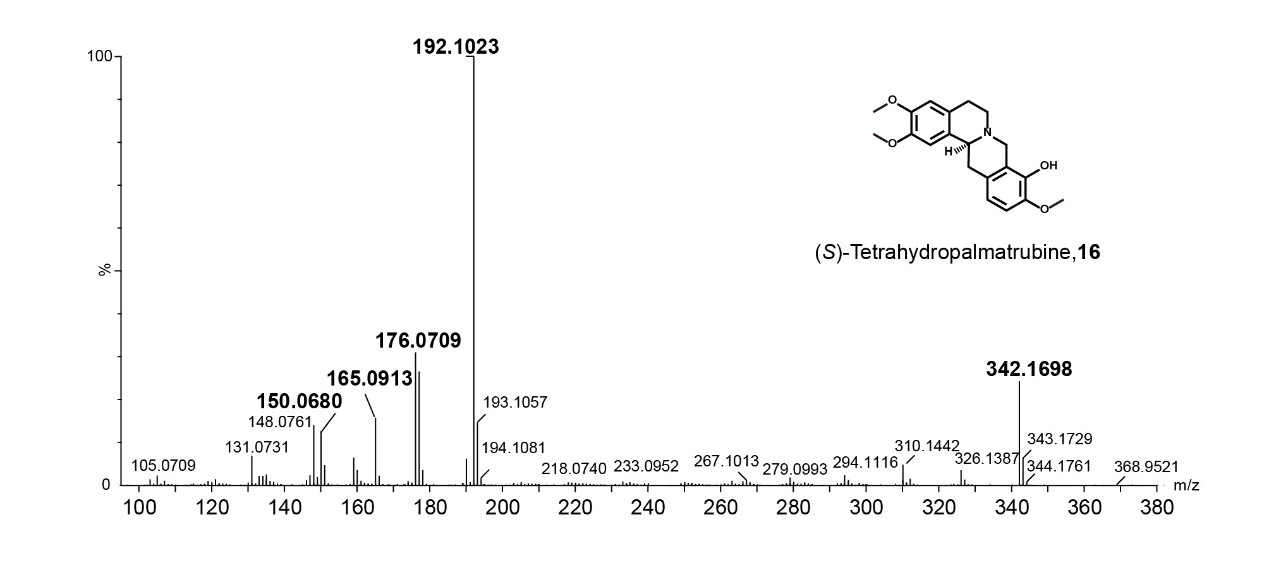


**Fig. S6** Key residues analysis of 11 genes clustered with 6OMTs clade in Fig. 3. Asterisks indicated four highly conserved candidate amino acids, and the arrows pointed to the seven key amino acids, which didn’t conserve in CyOMT5, PsN7OMT and St6OMT1, consistent with that the characterized functions of CyOMT5, PsN7OMT preferred to catalyze 7-*O*-methylation of 1-BIAs.


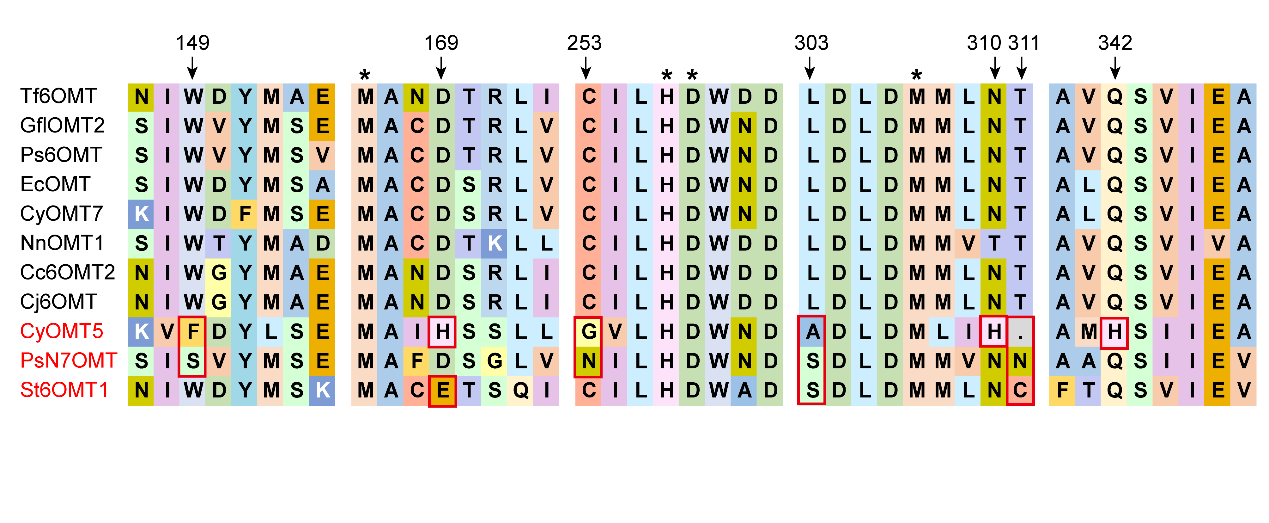


**Fig. S7** SDS-PAGE analysis for purified CyOMTs and mutants. Lane M: molecular weight marker; Lanes 1-11: purified enzymes of CyOMT2, CyOMT2_E169D, CyOMT2_L253C, CyOMT5, CyOMT5_F152W, CyOMT5_H172D, CyOMT5_G256C, CyOMT5_A306L, CyOMT5_H313N, CyOMT5_/314T and CyOMT5_H344Q, respectively.


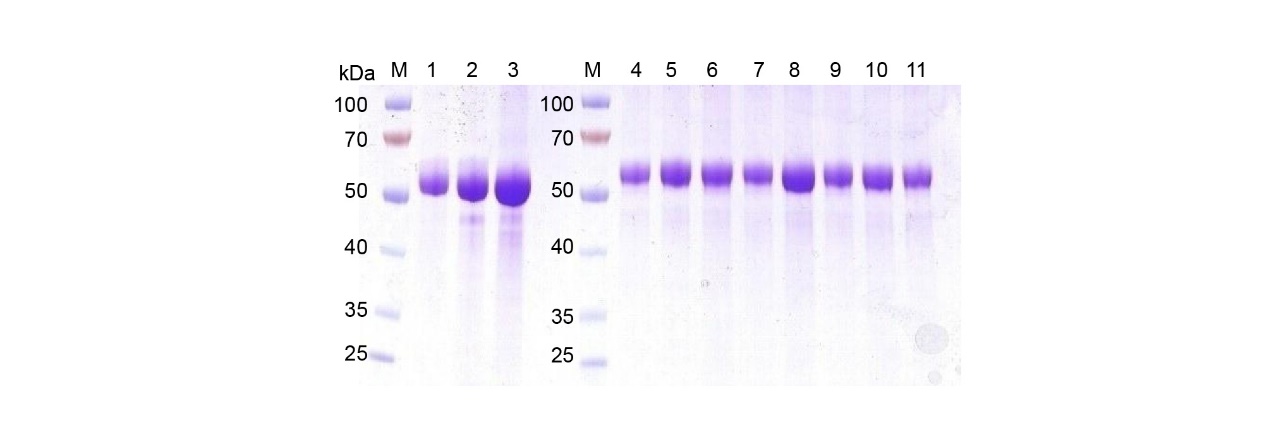


**Fig. S8** Effect of pH and temperature on the *O*-methylation activity of CyOMT2_L253C using norcoclaurine (**1**), scoulerine (**15**), tetrahydrojatrorrhizine (**11**) and jatrorrhizine (**14**) as substrates, respectively. Values represent the mean ± standard deviation of three independent measurements.


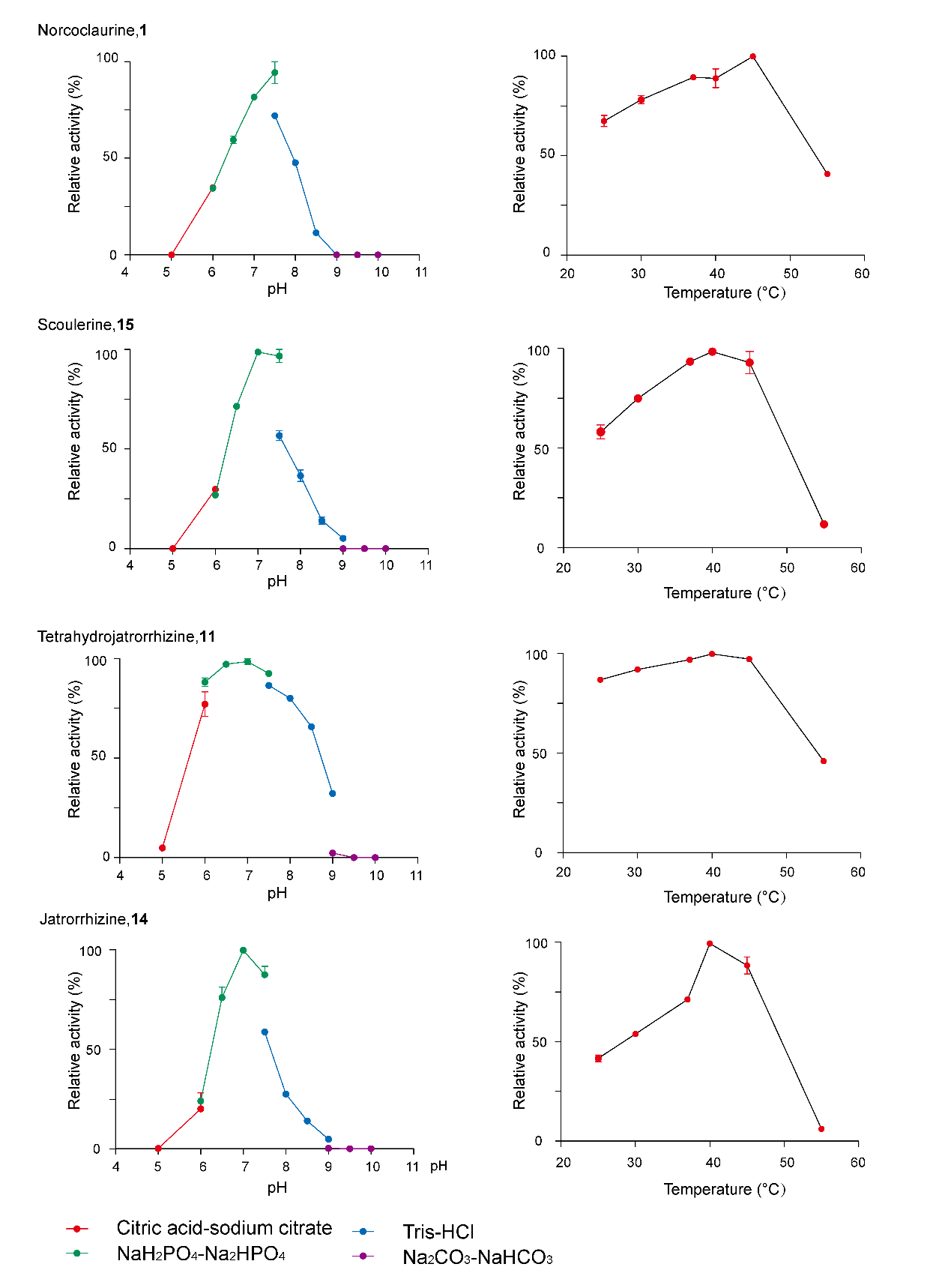


**Fig. S9** Kinetic parameters of purified CyOMT2 and CyOMT2_L253C using norcoclaurine (**1**), scoulerine (**15**), tetrahydrojatrorrhizine (**11**) and jatrorrhizine (**14**) as substrates with detection of their corresponding *O*-methylation activities. Values represent the mean ± standard deviation of three independent measurements.


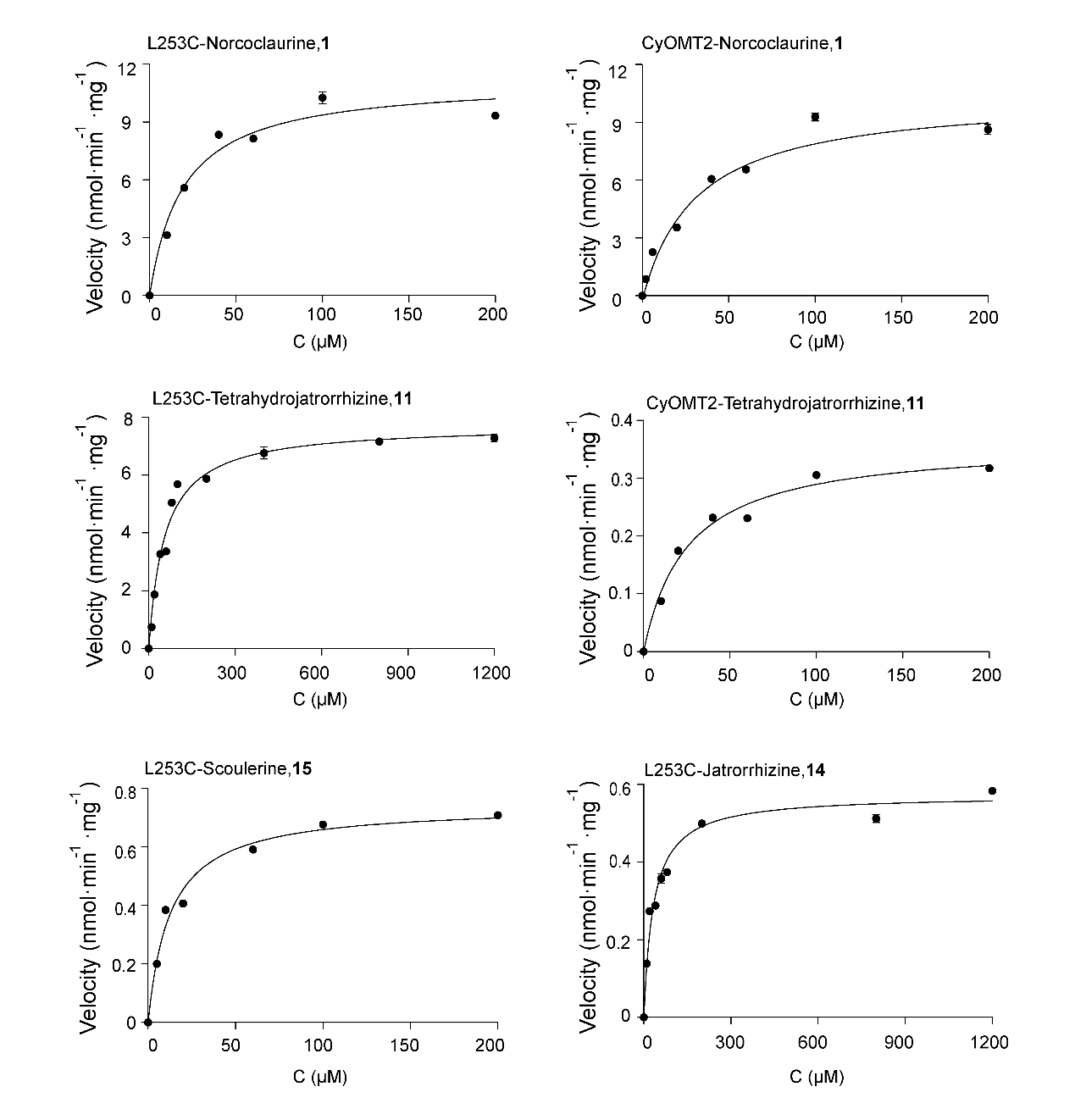


**Table S1.** Quality of the transcriptome data.

| **Sample** | **Raw Reads** | **Clean reads** | **Clean bases** | **Error (%)** | **Q20**  **(%)** | **Q30**  **(%)** | **GC**  **(%)** |
| --- | --- | --- | --- | --- | --- | --- | --- |
| YHS_L1 | 53253734 | 52758816 | 7.91G | 0.03 | 97.13 | 92.11 | 42.57 |
| YHS_L2 | 62656664 | 61775740 | 9.27G | 0.03 | 97.58 | 93.09 | 42.78 |
| YHS_B1 | 57074684 | 56562130 | 8.48G | 0.03 | 97.51 | 92.92 | 43.22 |
| YHS_B2 | 52038138 | 51566068 | 7.73G | 0.03 | 97.42 | 92.75 | 43.44 |

**Table S2.** The full-length sequences of *Corydalis yanhusuo* OMTs used for functionally characterized in this study.

| **Gene** | **mRNA sequence (5’-3’)** |
| --- | --- |
| CyOMT1 | ATGGCGAGATCACAGGATTCACAGCCACATGGAATAATATTGAATACGGATTCTTATCAATCATTCGAAGCTGAGGTTCAAGTTTATAATTATGCACTCAGCTTCATAAGCTCCATGTCCCTCATGTGTGCTGTTCAATTAGGGATACCGGACATCATTCACAATCATGGCAGCTGCAAGCCCATCACTTTGTCCAACCTTGTTGATGCTCTCTCATTGCCATCGACGAAAACCGAATATGTTCATCGTTTGATGCGCTTTTTGGTTCACATAGGCTGTTTTGGTCATGTGGATCAAGAAGGTTTTGTTCTCACACCCTTGTCAAGGCTCTTACTGACAGATGCTAATAACAGCTTGTCATCTTTCTTGCTTGGTATGCTAGATCCAGCTATGGTAACCCCTTGGCATTTCGTAAGCACTTGGCTTCGAGCTACTAGTAGTACTAATAATATTACTCCTAGCGGCGGTGCTGATGACGGTCTCATGCAAACTATGCC.TTTCGAGTTTGCCCATGGTATGAATATTTGGGACTTCATGAAGAAAAACTCTGATTTTAACAAAACCATCAATGAGGCCATGGCTTGTGACACTAGATTGGTGATGAATAGTGTGCTTAATGATCCCAAAGTAGGTGAGATGGTATTCAATAAGAATTTGACAACATTGATTGATGTAGGAGGCGGCATTGGAGCTGCGGCTATATCCATAGCTGAAGCATACCCACACTTGAAATGCTCTGTACTTGACCTCCCACATGTTGTTGCAGCTGCAACCGATGTACCGAGGAGCAACAACAATGCCGTTCACGTCATAGA.GTACATCGGTGGGGACATGTTTGAGTTCATCCCTCACACTGATGCTATATTTCTCAAGTATATACTTCATGACTGGAGTGATGAGAATTGTTTGAAGCTGTTGGGAAGATGCAAAGAAGCTATCCTTCCATCCAAAGAGAAAGGTGGGAAGCTGATTATAGTGGATATGGTGCTGGAAGACAAGAAAAAAGAACATGAAAGAACTCAAGCTCAGTATTTATTGGATGTGCTTATGATGGTAAACACCACAGGGAAAGAAAGAAATGAGAAACAATGGGAGTCTCTCTTCCTCAAATCTGGTTTCAATCACTACAAGATAACTTCTCTGGTAGGATTTAGGTCCCTCATTGAGGTCTATCCTTAA |
| CyOMT2 | ATGGGTGTCAATGATATAGCTGAAGCTCAGGATGTTGACATCAAAGCTCAAGCTCATCTATGGAATATAATCTATGGATTTGCCGATTCGCTTGTCCTTCGTTGCGCAGTTGAGCTCGGAATAGCAGACATTATCAATAGCAACAATGGGACAGTCACAATCTCCGACATTGCATCGAAACTCCCGGTTGACAATGTTAATGAAGAAAACTTATATCGGGTTTTACGATACTTAGTCTACATGGGTCTCTTGAAAGAGTCCCAAGATAAGTGTTACTCATTAGAACCAGTTGCTACTTTGCTCTTGAAAGATGCTCAGAGAAGTATGGTTCCTATCATTCTAGGAATGACTCAAAAGGATTTCATGGTTCCTTGGTTTTTCATGAAAGAAGGATTGGGTAGTGGGAGTACTACTGCATTTGAGAAAGGAATGGGAATGACTCTTTGGGAGTATTTGGAAGGACACCCAGATCAGAGTCAGTTGTTTAATGAAGGAATGGCTGGTGAAACTAGACTTTTGACTTCTTCGCTTATCAATGGTTGCCGTGATACTTTCCAAGGTTTAACTTCTCTTGTTGATGTTGGAGGAGGAAATGGTACAACCATTAAAGGTATTTACGATGCATTTCCACATATTAAATGCTCTGTGTATGATCTCCCTCACGTCATTGCTAATGCTCATCCTCATCCCAACATCGAGCGAATACCTGGAGATATGTTCAAGTCCGTTCCAAGTGCACAGGCAATTTTACTCAAGCTGATCTTGCACGACTGGACTGACGAAGAAAGTGTGGACATTCTAAAGAGATGCAGAGAAGCAGTACCTAAAGAAGGAGGAAGAGTGATTATCGTTGACGTAGCACTGGAGGAGGGATCTGAACATGAGCTTACGAAGACGAGATTGATACTTGATATTGATATGTTGGTGAACACAGGTGGGAGAGAAAGGACTGTTGATGATTGGGACAGAATGCTCAAACTTGCAGGGTTTAGTAGTCACAAGATCAGGCACATTGCAGCCATTCAATCTGTCATTGAAGCCTTCCCATAG |
| CyOMT3 | ATGGGTTCAACTGAGAATGAAATCAAGACCAGTGCTACTACCCCAGAAGAAGAAGAAGAAGCATGTTTGTATGCTATGCAATTAGCAAGTGCTTCAGTTCTTCCCATGATACTTAAATCAGCCATAGAACTTGATTTGCTTGAAATCATGGCTAAATCTGGTGTGGGTGCCTATATAAGTCCATCTGAGCTTGCTTCTAAGCTTCCTACCACAAACCCTGCTGCTCCTGTGATGCTTGACCGTATGCTCCGTCTCTTGGCGAGTTACAAAGTTCTCACGTGTAAACTTAACACGCTTGATGACGGTCGAGTCGAAAGGTTATATGGGCTTGCACCCGTTTGCAAGTACTTGATTAAGAATGAAGATGGTGTGTCCATGGCTCCTCTGGTCCTCATGAATCAAGACAAAGTCCTCATGGAGAGCTGGTACTACTTGAAGGATACAGTTCTTGATGGTGGAATTCCATTTAACAAAGCTTATGGAATGACCGCATTCGAATACCACGGTACAGATCCCAGATTCAACAAGGTTTTCAACAGAGGAATGTCTGATCATACTACCATTACAATGAAGAAGATTCTTGAGACATACAAGGGATTCGAAGGTTTGAATTCCATCGTAGATGTTGGTGGTGGTATCGGTGCCACAGTTAGTATGATCGTCTCTAAGTACCCGTCTATTAAAGGGATTAACTTCGACTTGCCTCATGTCATTGAAGATGCACCGACTTATCCCGGTGTTGAGCATGTTGGAGGAGACATGTTTGCTAGTGTCCCAAAAGGGGATGCCATTTTCATGAAGTGGATTCTTCATGACTGGAGTGATGAACACAACCTGAAAACTTTGAAGAACTGTTATGAAGCGTTGCCTGCCAATGGTAAGGTGATCATTGCGGAATCAATTCTTCCGGTGTTCCCTGAAACTAATGCTGCAGCTAGCGGTGTTTTCCATGTCGATGTCATCATGTTGGCACACAACCCTGGTGGAAAAGAAAGAACCGAGAAAGAGTTCGAGGCCTTAGCAAAGGAAGCTGGGTTTTCCGGTTTTAAAGTTGTTTGCTGTGCTTATAACAGTTGGATCATGGAATTCTGTAAATAG |
| CyOMT4 | ATGGCCATGGGATCATCACCACAAGGAACAATATTGAAGGATTCTTATGATCAGTTATTGGGAGATGAGGTTCAAGTTTATAACTATGCATTGAGCTTCATAAGCTCCATGTCCCTCAAGTGTGCTGTTCAATTAGGGATACCGGACATCATTCACAATCATGGCAGCAGCAAACCCATCACTTTGTCCAATCTTGTTGATGCTCTCTCACTGCCATCGACGAAAACCGAATATGTTCATCGCTTGATGCGCTTTTTGGTTCACATAGGTTGTTTCGCTAATGTGGAAGAAGGTTATGTGCTCACACCCTTGTCAAGGCTCTTACTAAAAGATGCTAATAACAACTTGTCATCTTTCTTGCTTGGTATGCTAGATCCAGTTCTGGTAAGCCCTTGGCATTTCATAAGCACTTGGCTTCGAGCTAGTAGTAGTACTAATAATAGTACTCCTAGCGGCGGTGGCGATGATGCTCTCATGCAAACGACGCCATTCGAGTTGGCTCATGACGGCATGAATATGTGGGACTTCATGGAGAAAAACTCTGAATTTAACAAAATCTTCAATGAGGCCATGGCTTGTGACACTAGGTTTGTGATGAATGGTGTAATTCATGACCCCAAAGTAGGTGAGATGGTATTCAATAAGAATTTGACAACATTGATTGATGTAGGAGGTGGAATCGGAGCTGCGGCCAGATCCATTACTGAAGCATACCCACATTTGAAATGCTCGGTACTTGACCTCCCACATGTGGTTGCAGCAGCAACCGATGTGCAGAGGAACAACAACAATATTGCCGTCGACGTCATAGAGTACATTGGTGGGGACATGTTTGAGTTCATCCCTCACACTGATGCTATATTTCTCAAGTATATTCTTCATGACTGGAGTGATGAGAGTTGCTTGAAGATATTGGGAAGATGCAGAGAAGCTATCCTTCCATCCAAAGAGAAAGGTGGAAAGCTGATTATAGTAGATATAGTGGTGGAAGACAGGAAAGAAGAACATGAGAGAACACAAATCCAGTATATGTTTGATATGGTGATGATGGTCGAAACCTCAGGGAAAGAAAGAAATGAGAAACAATGGGAGTATCTCTTCCTCAAATCTGGTTTCACTCACTACAAGATAACTTCTCTTGTGGGAGCTAGGTCCCTCATTGAGGTATATCCTTAA |
| CyOMT5 | ATGGAAGGGATGAGTGATCTATCCCTTGAAAACGAAGCCGTAACAATCTGGAAATTCATCTACGGATTTTGTGATACACTAGTTCTAAAATGTTGTGTAAACCTTGAAATAGCCGATACAATTCATAAGCATGGCCAACCTATGACACTCTCCGAATTAGCTTCTCAACTTTCTGTTGACCTCCAAAAAACCATTGATACCGACCGTCTATATCGATTGATGCGGTATCTGGTTCACTTGAAGTTCTTTACAACAGAAGAAGGATCTGATCATTTAGGGGAAATCAAGTATGGGCGGTTGCCACTGGCAAAATTCTTAATCAGAGGATGGCCGAAGTCCATGGCTGGTTTGTTAACAGCTATGGATAAGGATTTCATTGCACCTTGGCATCATCTCGAGGATGGTTTGGATGGTCGCACCGACGCTTTCGAAGTAGCATTTGGGAAGAAAGTTTTTGATTATTTGTCCGAAAACCCCAAAAGCAGTCAGCTTTTCAATGATTATATGGCTATTCATAGTAGTCTCCTTGCTTTGGAGTTGGTTAAGTGTAAGAATGTTTTTGAAGACCGGATTAAAACACTTGTTGATGCTGGTGGTGGCACCGGAACAACTGCTAAAGCAATTGCCAACGCTTTCCCACACATAAAATGCATGGTTTATGAACTTCCTCACGTGAATGCGGATGCTCCCGTTGATCCTAATATCCAACGAATCGATGGGGATATTTTCAAGTCTGTTCCCAAGGCAGATGCAATTTTAATGCAGGGGGTCCTCCACGATTGGAATGACGACGAATGTATTCAAATATTAAAGAATTGCAGAGAATCAATACCCCAAGATGGAGGGAAAGTTATCATCATCGAAGTCGTGGTGAATGCGAATTCGAAACATCCTTATTCAAAAATTATGTTATTGGCGGATTTAGATATGTTGATCCACGGAGGGAGGGAGAGAACCGACGAGGAGTGGAAGAAACTACTCGAAGCTGCTGGTTTCACTAGATACAAACTCACTGAAATATCGGCGATGCATTCAATAATTGAAGCTTATCCCTATTAA |
| CyOMT6 | ATGGATGCTCCAGTAAACTATCTTTCTGGATTTGAGTTATTGTCTCGACTGACATGCTTTCCAATGGCATTACGTGCTGCAATCGATCTAAACGTGTTTCCAATCATCTCAGGTTTTGGACTCGATGCAAAACTTAGTGCATCACAGTTGGTAGCCCAGATGCCAACGACTAACCCGAATGCTGCCACTGCTTTGGAACGAATCCTTAGAATCCTTGCAGCAAACTCCCTTTTGTCACCGTCTAATGAACTTAACGGCGAAATTTCTTATGGATTAACAGAAGATTCGCGATACTTGGTTCCCGTCCAGAAAGATGGTGTTTCTCTTGTGCCAATGGTGTTGTTGACGATTAATAAGTATGTTATGGAGAGCTTCTTTCAGCTTAAGGATGCTGTGCTTGATGAAGGTTGTGTGCCTTTTGATAGAACTTTTGGTGTAAGTATCTTTGAGTTTGCTGGGAAGGAACCAAAAGTGGGTAACATTTTTAATGAAGCGATGAGGTCTAGCTCTAGTTTCGTTCTGGATGAGGTTTTTAAGGTTTACGAGGGTTTTGATGAAATGAAGGAGTTGGTTGATGTTGGTGGTGGTATTGGAGGTACAATGAGTAAGATTGTTTCCAAGTTTTCTCACATTCATGGTATCAACTTTGATTTGCCTCATGTTATTGTCGATGCTCCAAATTATCCGGGTGTGAAGCATATCTCTGGAGATATGTTTGAAGAAATACCCAAGGCAGAAAACATATTTCTTAAGTGGGTACTTCATGATTGGGATGATGAAAGTTGCAAGAAATTGCTAAGGAAATGCTGGAATGCATTGGATGAAGGTGGAAAGGTGATAGTAATAGAGTTGGTGCTACCTCAAGTACTAGGAAACAATGCAGAGTCTCATAGTGCTTTAGCCGGTGATCTCATGATGATGGCATTGAGTCCTGGCGGAAAAGAGAGAACAATAATTCAGTTTCACAACCTAGCCCAAGCAGCTGGATTCAACAAAGTCAAGTCTTTCCCAGTCAACCAAGGTCTCCATGTCATTGAATTCCAAAAATAG |
| CyOMT7 | ATGGAAGTGATCAAGAAGAGTGATCAAACAGATCAAGCCAAACTCTGGAAGTTCATCTATGGATTTGCAGATTCACTAGTTCTTAAATGCGCGGTGGAGCTAGAAATAGCCGATACGATTCATAAGCATGGGGAACCGATGACGCTTTCCGAATTAGCTTCTCAACTTCCTAAGCAACCTATCGATGCAGACCGTCTATATCGAATAATGCGGTACTTGGTTCAAATAAAATTGTTTAGCAAAGAGACGACTTCTGAATCCGGGGAAATCAAATACGGGCTTTTACCACCGGCGAAATATGTGGTAAGAGGATGGCAGAATTCCATGGTTGCTGCATTGCTATTAATCAATGATAAGAATTTCATTGCATCTTGGCATTATCTCAAGGATGGTTTGGGTGGCGAATGTGATGCATTTGAGAAGGCTAACGGAAAGAAAATTTGGGATTTTATGTCCGAAAACCCCGAAAAGAATAAACTTTTCAATGAGGCTATGGCTTGTGATAGTAGGCTCGTTACTTGGGCGTTGGTTCAAGATTGTAAGGATGTTTTCAAAGGAATTAAGACACTTGTTGATGTTGGTGGTGGCACTGGAACCGCAGTGAAGGCGATTTCTGATGCTTTTCCGGATATAAAATGCGCGGTTTATGATCTTCCTCATGTCATTGCGGATTCTCCAGTTGCTCCTAATATTGATCGAATCGAGGGGGATATGTTTAAGTCCATCCCAAATGCAGATGCCATCTTTATGAAGTGCATCCTCCATGATTGGAACGACGACGAATGCATTCAAATACTTAAGCAATGTAAAAAGGCGCTACCACGAGACGGAGGTAAAGTAATCATCGTAGATGTCGTGTTGAATGTGGATTCGAAGCATCCTTACACAAAAATGAGATTGACTTTGGATTTGGATATGATGCTCAACACTGGAGGGAAAGAGAGGACAGAGGAGGAATGGAAAGAACTGTTTGAAGCTGCAGGTTTCAGTGGATACAAAATCATTCAAACATCAGCACTACAATCTGTGATTGAGGCTTATCCTTAA |

**Table S3.** Primers used for full-length gene cloning and real-time PCR.

| **Genes** | **Primers (5’-3’) (Full-length gene cloning)** |
| --- | --- |
| CyOMT1-F | GCCATGGCTGATATCGGATCCATGGAAGTGACCAAGAGTGA |
| CyOMT1-R | ACGGAGCTCGAATTCGGATCCTTAACCAGGAAATGCCTCTATC |
| CyOMT2-F | GCCATGGCTGATATCGGATCCATGGGTGTCAATGATATAGC |
| CyOMT2-R | ACGGAGCTCGAATTCGGATCCCTATGGGAAGGCTTCAATG |
| CyOMT3-F | GCCATGGCTGATATCGGATCCATGGGTTCAACTGAGAATG |
| CyOMT3-R | ACGGAGCTCGAATTCGGATCCCTATTTACAGAATTCCATGATC |
| CyOMT4-F | GCCATGGCTGATATCGGATCCATGGCCATGGGATCATCACC |
| CyOMT4-R | ACGGAGCTCGAATTCGGATCCTTAAGGATATACCTCAATGAGGG |
| CyOMT5-F | GCCATGGCTGATATCGGATCCATGGAAGGGATGAGTG |
| CyOMT5-R | ACGGAGCTCGAATTCGGATCCTTAATAGGGATAAGCTTC |
| CyOMT6-F | GCCATGGCTGATATCGGATCCATGGATGCTCCAGTAAACTATC |
| CyOMT6-R | ACGGAGCTCGAATTCGGATCCCTATTTTTGGAATTCAATGACATG |
| CyOMT7-F | GCCATGGCTGATATCGGATCCATGGAAGTGATCAAGAAGAG |
| CyOMT7-R | ACGGAGCTCGAATTCGGATCCTTAAGGATAAGCCTCAATCAC |
| **Genes** | **Primers (5’-3’) (Recombinant plasmid cloning)** |
| CyOMT5-F149W-F | TTTTGGGGTTTTCGGACAAATAATCCCAAACTTTCTTCCCAAATGCTACTTC |
| CyOMT5-F149W-R | GAAGTAGCATTTGGGAAGAAAGTTTGGGATTATTTGTCCGAAAACCCCAAAA |
| CyOMT5-H169D-F | TCCAAAGCAAGGAGACTACTATCAATAGCCATATAATCATTGAAAA |
| CyOMT5-H169D-R | TTTTCAATGATTATATGGCTATTGATAGTAGTCTCCTTGCTTTGGA |
| CyOMT5-G253C-F | CATTCCAATCGTGGAGGACGCACTGCATTAAAATTGCATCT |
| CyOMT5-G253C-R | AGATGCAATTTTAATGCAGTGCGTCCTCCACGATTGGAATG |
| CyOMT5-A303L-F | CCCTCCGTGGATCAACATATCTAAATCTAGCAATAACATAATTTTTGAATAAGGATGTTTCG |
| CyOMT5-A303L-R | CGAAACATCCTTATTCAAAAATTATGTTATTGCTAGATTTAGATATGTTGATCCACGGAGGG |
| CyOMT5-H310N-F | TGATCAACATATCTAAATCCGCCAATAACATAA |
| CyOMT5-H310N-R | GATTTAGATATGTTGATCAACGGAGGGAGG |
| CyOMT5-/311T-F | GGTGTGGATCAACATATCTAAATCCGCCAATAACATAA |
| CyOMT5-/311T-R | TAGATATGTTGATCCACACCGGAGGGAGGGAG |
| CyOMT5-H342Q-F | GATAAGCTTCAATTATTGACTGCATCGCCGATATTTCAGTGAGTTTG |
| CyOMT5-H342Q-R | CAAACTCACTGAAATATCGGCGATGCAGTCAATAATTGAAGCTTATC |
| CyOMT2-E169D-F | GAAGAAGTCAAAAGTCTAGTATCACCAGCCATTCCTTCATTAAA |
| CyOMT2- E169D-R | TTTAATGAAGGAATGGCTGGTGATACTAGACTTTTGACTTCTTC |
| CyOMT2-L253C-F | GTCAGTCCAGTCGTGCAAGATGCACTTGAGTAAAATTGCCTGTGCA |
| CyOMT2-L253C-R | TGCACAGGCAATTTTACTCAAGTGCATCTTGCACGACTGGACTGAC |
| **Genes** | **Primers (5’-3’) (Real-Time PCR)** |
| CyOMT1-F | TATGGTGCTGGAAGACAA |
| CyOMT1-R | AGGGACCTAAATCCTACCAG |
| CyOMT2-F | GAAGGATTGGGTAGTGGG |
| CyOMT2-R | GATAAGCGAAGAAGTCAAA |
| CyOMT3-F | CTGCCAATGGTAAGGTGA |
| CyOMT3-R | AACCCAGCTTCCTTTGCT |
| CyOMT4-F | AAACCCATCACTTTGTCC |
| CyOMT4-R | ATGCCAAGGGCTTACCAG |
| CyOMT5-F | GGAATGACGACGAATGTA |
| CyOMT5-R | TTCCACTCCTCGTCGGTTC |
| CyOMT6-F | AGTTGGTAGCCCAGATGC |
| CyOMT6-R | AACAACACCATTGGCACA |
| CyOMT7-F | AAGGCGCTACCACGAGAC |
| CyOMT7-R | GATAAGCCTCAATCACAGA |
| UBQ-F | TCTGCATACCTCCACGAA |
| UBQ-R | GCCAAGATCCAGGACAAG |

**Table S4**. Abbreviations and GenBank accessions numbers for functionally characterized plants OMTs used for phylogenetic analysis.

| Gene | SOURCE | Accession |
| --- | --- | --- |
| Ps6OMT | *Papaver somniferum* | AY217335 |
| Ps7OMT | *Papaver somniferum* | AY268893 |
| Ps4’OMT | *Papaver somniferum* | AY217333 |
| Ps4’OMT2 | *Papaver somniferum* | AY217334 |
| PsN7OMT | *Papaver somniferum* | FJ156103 |
| PsSOMT1 | *Papaver somniferum* | JN185323 |
| Cc6OMT2 | *Coptis chinensis* | MH165876 |
| Ct7OMT | *Coptis teeta* | MH165877 |
| CtSOMT | *Coptis teeta* | MH165874 |
| GflOMT1 | *Glaucium flavum* | KP176693 |
| GflOMT2 | *Glaucium flavum* | KP176694 |
| GflOMT6 | *Glaucium flavum* | KP176698 |
| GflOMT7 | *Glaucium flavum* | KP176699 |
| NnOMT1 | *Nelumbo nucifera* | XM_010245752 |
| NnOMT5 | *Nelumbo nucifera* | XM_010277761 |
| Cj6OMT | *Coptis japonica* | D29811 |
| Cj4’OMT | *Coptis japonica* | D29812 |
| CjCoOMT | *Coptis japonica* | AB073908 |
| CjSOMT | *Coptis japonica* | D29809 |
| EcOMT | *Eschscholzia californica* | AB745042 |
| Ec4’OMT | *Eschscholzia californica* | AB745041 |
| Tf4’OMT | *Thalictrum flavum* | AY610510 |
| TfSOMT | *Thalictrum flavum* | AY610512 |
| Tf6OMT | *Thalictrum flavum* | AY610507 |
| St6OMT1 | *Stephania tetrandra* | Cef^2^ |
| St6OMT2 | *Stephania tetrandra* | Cef^2^ |
| HNMC4’OMT | *Sinopodophyllum hexandrum* | KJ786960 |
| GsOMT1 | *Gloriosa superba* | MT512039 |
| GsOMT2 | *Gloriosa superba* | MT512043 |
| GsOMT3 | *Gloriosa superba* | MT512044 |
| GsOMT4 | *Gloriosa superba* | MT512046 |
| ObCVOMT | *Ocimum basilicum* | AAL30423 |
| OtEuOMT | *Ocimum tenuiflorum* | EU622049 |
| ZmCOMT | *Zea mays* | NP_001106047 |
| RhcOMT | *Rosa hybrid cultivar* | AAM23004 |
| CbCOMT | *Clarkia breweri* | AAB71141 |
| CbSAMT | *Clarkia breweri* | AF133053 |
| CbIEMT | *Clarkia breweri* | AAC01533 |
| EglCOMT | *Eucalyptus globulus* | AAD50440 |
| CaCOMT | *Chrysosplenium americanum* | AAA86982 |
| Ca3’FOMT | *Chrysosplenium americanum* | AAA80579 |
| Sl3’5’FOMT | *Solanum lycopersicum* | NP001289828 |
| TaFOMT2 | *Triticum aestivum* | ABP63535 |
| TaFOMT | *Triticum aestivum* | Q84N28 |
| VvAOMT | *Vitis vinifera* | C7AE94 |
| VvCCoAOMT | *Vitis vinifera* | CAA90969 |
| VvROMT | *Vitis vinifera* | PPTB6VJS4 |
| HvF7OMT | *Hordeum vulgare* | CAA54616 |
| MsI7OMT | *Medicago sativa* | U97125 |
| MsChOMT | *Medicago sativa* | AAB48059 |
| MsCOMT | *Medicago sativa* | AAB46623 |
| MsCCoAOMT | *Medicago sativa* | AAC28973 |
| MtHI4’OMT | *Medicago truncatula* | AY942158 |
| GeHI4’OMT | *Glycyrrhiza echinata* | AB091684 |
| PtCOMT | *Pinus taeda* | AAC49708 |
| PtCCoAOMT | *Populus tremuloides* | AAA80651 |
| NtCCoAOMT | *Nicotiana tabacum* | AAC49913 |
| HlOMT2 | *Humulus lupulus* | B0zb56 |
| AtJMT | *Arabidopsis thaliana* | Q9AR07 |
| AtIAMT | *Arabidopsis thaliana* | Q9FLN8 |
| RgOMT | *Ruta graveolens* | AY894417 |
| SoCOMT | *Saccharum officinarum* | O82054 |
| ZeCOMT | *Zinnia elegans* | Q43239 |
| EgCOMT | *Eucalyptus gunnii* | P46484 |
| Os3’FOMT | *Oryza sativa* | Q6ZD89 |
| PsHM3OMT | *Pisum sativum* | AAC49856 |
| McPFOMT | *Mesembryanthemum crystallinum* | AAN61072 |
| ShOMT1 | *Sinopodophyllum hexandrum* | KT390155 |
| LAMT | *Catharanthus roseus* | KF415116 |
| ObF8OMT | *Ocimum basilicum* | KC354402 |
| MpF7OMT1 | *Mentha x piperita* | AAR09597 |
| MpF7OMT2 | *Mentha x piperita* | AAR09598 |
| MpF4’OMT | *Mentha x piperita* | AY337461 |
| MpF8OMT | *Mentha x piperita* | AY337459 |
| MpF3’OMT | *Mentha x piperita* | AY337460 |
|  |  |  |

**Table S5**. Chromatographic and mass spectral information for those compounds used in this study.

| **Alkaloid** | **Formula** | ***m/z***  **[M+H]^+^** | **Rt**  **(min)** | **Product ions *m/z***  **(Relative abundance, %)** |
| --- | --- | --- | --- | --- |
| (*S*)*-*norcoclaurine (**1)** | C_16_H_17_NO_3_ | 272.1289 | 2.49 | 272(9);255(5);194(23);165(15),143(13);  123(8);115(25);107(100) |
| (*S*)*-*coclaurine (**2**) | C_17_H_19_NO_3_ | 286.1445 | 3.41 | 286(8);269(3);254(3);237(3);209(7);194  (25);165(20);143(12);115(25);107(100) |
| (*S*)*-*3’-hydroxy-*N*-methylcoclaurine (**6**) | C_18_H_21_NO_4_ | 316.1555 | 3.00 | 316(19);298(10);255(4);225(6);207(15);192  (54);177(51);160(11);143(44);137(16);123  (100);115(32); |
| (*S*)*-*reticuline (**7**) | C_19_H_23_NO_4_ | 330.1720 | 3.97 | 330(28);299(3);269(3);235(5);207(13);192(100);177(75);143(44);137(83);122(14);115(23);91(4) |
| norarmepavine (**4**) | C_18_H_21_NO_3_ | 300.1609 | 4.36 | 300(2);282(2);268(8);252(7);237(18);225(4);194(9); 174(10);145(14);131(10);107(100) |
| (*S*)*-*scoulerine (**15**) | C_19_H_21_NO_4_ | 328.1569 | 4.32 | 328(38);296(4);252(2);178(100);163(69);  151(11);135(8);119(6);91(3) |
| (*S*)*-*tetrahydro  columbamine (**9**) | C_20_H_23_NO_4_ | 342.1723 | 5.06 | 342(15);326(2);178(100);163(59);151(8);  135(6);119(3) |
| (*S*)*-*tetrahydro  palmatine (**10**) | C_21_H_25_NO_4_ | 356.1859 | 6.06 | 356(15);340(3);322(1);308(1);192(100);177(23);165(11);148(11);131(5);105(2) |
| tetrahydro  jatrorrhizine (**11**) | C_20_H_23_NO_4_ | 342.1719 | 5.21 | 342(16);326(2);178(100);163(62);151(6);  135(5);119(3) |
| jatrorrhizine (**14**) | C_20_H_20_NO_4_ | 338.1408 | 6.16 | 338(2);322(82);307(75);294(49);279(100);  265(63);250(20);237(19);222(7);192(1); |
| columbamine (**12**) | C_20_H_20_NO_4_ | 338.1398 | 6.04 | 338(2);322(85);307(78);294(53);279(100);  265(63);251(20);237(15);222(7);208(1) |
| palmatine (**13**) | C_21_H_21_NO_4_ | 352.1564 | 7.00 | 352(2);336(100);320(78);308(54);304(24);292(87);278(58);275(13);264(17);250(15);  233(6);220(2);191(1) |

**Table S6.** Quality control of the homology modeling of CyOMTs with SWISS-MODEL. Model index include sequence similarity with template Tf6OMT, GMQE value, clash score, Ramachandran score (%) and molprobity score of CyOMT2, CyOMT5, CyOMT7, respectively.

| **Model index** | **CyOMT2** | **CyOMT5** | **CyOMT7** |
| --- | --- | --- | --- |
| **Sequence similarity (%)** | 53.71 | 53.10 | 67.15 |
| **GMQE value** | 0.81 | 0.81 | 0.86 |
| **clash score** | 2.09 | 4.57 | 2.47 |
| **Ramachandran score (%)** | 98.21 | 98.54 | 98.26 |
| **molprobity score** | 0.98 | 1.23 | 1.03 |

**REFERENCES**

1. Valentic TR, Payne JT, Smolke CD. Structure-Guided Engineering of a Scoulerine 9-*O*-Methyltransferase Enables the Biosynthesis of Tetrahydropalmatrubine and Tetrahydropalmatine in Yeast. *ACS Catalysis* 2020; **10**(8)**:** 4497-4509.

2. Li Q, Bu J, Ma Y, Yang J, Hu Z, Lai C*, et al.* Characterization of *O*-methyltransferases involved in the biosynthesis of tetrandrine in Stephania tetrandra. *J Plant Physiol* 2020 Jul; **250:** 153181.
